# Establishing Healthcare Worker Performance and Safety in Providing Critical Care for Patients in a Simulated Ebola Treatment Unit: Non-Randomized Pilot Study

**DOI:** 10.3390/v13112205

**Published:** 2021-11-02

**Authors:** Peter Kiiza, Sarah I. Mullin, Koren Teo, Len Goodman, Adic Perez, Ruxandra Pinto, Kelly Thompson, Dominique Piquette, Trevor Hall, Elhadj I. Bah, Michael Christian, Jan J. Hajek, Raymond Kao, François Lamontagne, John C. Marshall, Sharmistha Mishra, Srinivas Murthy, Abel Vanderschuren, Robert A. Fowler, Neill K. J. Adhikari

**Affiliations:** 1 Department of Critical Care Medicine, Sunnybrook Health Sciences Centre, Toronto, ON M4N 3M5, Canada; kiizaptr@gmail.com (P.K.); adic.perez@gmail.com (A.P.); ruxandra.pinto@sunnybrook.ca (R.P.); 2 Graduate Department of Psychological Clinical Science, University of Toronto, Toronto, ON M1C 1A4, Canada; sarah.mullin@mail.utoronto.ca; 3 Canadian Forces Health Services Group (CFHS), Toronto, ON M3K 0A1, Canada; koren.lui@mail.utoronto.ca; 4 Defence Research and Development Canada, Toronto Research Centre, Toronto, ON M3K 2C9, Canada; len.goodman@drdc-rdds.gc.ca; 5 The George Institute for Global Health, University of New South Wales, Newtown, NSW 2042, Australia; kthompson@georgeinstitute.org; 6 Department of Critical Care Medicine, Sunnybrook Health Sciences Centre, Interdepartmental Division of Critical Care Medicine, University of Toronto, Toronto, ON M4N 3M5, Canada; dominique.piquette@sunnybrook.ca; 7 Healthcare Insurance Reciprocal of Canada and Interactive Media Lab., University of Toronto, Toronto, ON M2N 6K8, Canada; thall@hiroc.com; 8 Infectious Diseases Department, Donka National Hospital, Conakry, Guinea; elbah9@hotmail.com; 9 Island Health Authority, Comox, BC V9M 1P2, Canada; michael.christian@islandhealth.ca; 10 Division of Infectious Diseases, University of British Columbia, Vancouver, BC V5Z 1M9, Canada; janjhajek@gmail.com; 11 Division of Critical Care Medicine, Western University, London, ON N6A 5W9, Canada; Raymond.Kao@lhsc.on.ca; 12 Department of Medicine, Université de Sherbrooke, and Centre de recherche du CHU de Sherbrooke, Sherbrooke, QC J1H 5N4, Canada; francois.lamontagne@usherbrooke.ca; 13 Departments of Surgery and Critical Care, St. Michael’s Hospital, Department of Surgery, Interdepartmental Division of Critical Care Medicine, University of Toronto, Toronto, ON M5B 1W8, Canada; john.marshall@unityhealth.to; 14 Li Ka Shing Knowledge Institute, Department of Medicine, Division of Infectious Diseases, St. Michael’s Hospital and University of Toronto, Institute of Health Policy, Management and Evaluation and Institute of Medical Science, University of Toronto, Toronto, ON M5B 1W8, Canada; sharmistha.mishra@utoronto.ca; 15 Department of Paediatrics, University of British Columbia, Vancouver, BC V6H 3V4, Canada; srinivas.murthy@cw.bc.ca; 16 Division of Intensive Care, Université Laval-CHU de Québec, Québec, QC G1J 1Z4, Canada; abel.vanderschuren.1@ulaval.ca; 17 Department of Critical Care Medicine, Sunnybrook Health Sciences Centre, Interdepartmental Division of Critical Care Medicine and Institute for Health Policy, Management, and Evaluation, University of Toronto, Toronto, ON M4N 3M5, Canada

**Keywords:** Ebola Virus Disease, Ebola Treatment Unit, simulation, personal protective equipment, critical care, critical illness

## Abstract

Improving the provision of supportive care for patients with Ebola is an important quality improvement initiative. We designed a simulated Ebola Treatment Unit (ETU) to assess performance and safety of healthcare workers (HCWs) performing tasks wearing personal protective equipment (PPE) in hot (35 °C, 60% relative humidity) or thermo-neutral (20 °C, 20% relative humidity) conditions. In this pilot phase to determine the feasibility of study procedures, HCWs in PPE were non-randomly allocated to hot or thermo-neutral conditions to perform peripheral intravenous (PIV) and midline catheter (MLC) insertion and endotracheal intubation (ETI) on mannequins. Eighteen HCWs (13 physicians, 4 nurses, 1 nurse practitioner; 2 with prior ETU experience; 10 in hot conditions) spent 69 (10) (mean (SD)) minutes in the simulated ETU. Mean (SD) task completion times were 16 (6) min for PIV insertion; 33 (5) min for MLC insertion; and 16 (8) min for ETI. Satisfactory task completion was numerically higher for physicians vs. nurses. Participants’ blood pressure was similar, but heart rate was higher (*p* = 0.0005) post-simulation vs. baseline. Participants had a median (range) of 2.0 (0.0–10.0) minor PPE breaches, 2.0 (0.0–6.0) near-miss incidents, and 2.0 (0.0–6.0) health symptoms and concerns. There were eight health-assessment triggers in five participants, of whom four were in hot conditions. We terminated the simulation of two participants in hot conditions due to thermal discomfort. In summary, study tasks were suitable for physician participants, but they require redesign to match nurses’ expertise for the subsequent randomized phase of the study. One-quarter of participants had a health-assessment trigger. This research model may be useful in future training and research regarding clinical care for patients with highly infectious pathogens in austere settings.

## 1. Introduction

Ebola Virus Disease (EVD) is a contagious zoonotic infection with case-fatality rates of up to 90% in humans [1,2]. Since the first reported Ebola outbreak in the Democratic Republic of Congo (DRC) in 1976, there have been 31 outbreaks [2], with two recent outbreaks in DRC and Guinea [3]. The largest EVD outbreak to date was in 2014–2016 in West Africa, with 28,652 cases and 11,325 deaths, corresponding to a case-fatality rate of 39.5% [2]. A recent completed EVD outbreak in the DRC had a case-fatality rate of 42% (55 deaths among 130 confirmed cases) [3]. Furthermore, EVD outbreaks curtail access to other essential health services and likely increase mortality for endemic conditions [4].

Over the past five decades, the prevention and control of EVD outbreaks have relied upon disease surveillance, infection prevention and control in health facilities and communities, risk communication, social mobilization, case management [2], and more recently, vaccination of high-risk groups [5] and EVD-specific investigational therapies [6,7]. Despite these strategies, EVD case-fatality rates remain at 40–60% in Africa [2], compared to 19% in well-resourced countries [8]. The provision of critical care to EVD patients in Africa, despite potentially reducing mortality [8,9,10,11], is challenging due to limited resources, a lack of available healthcare workers (HCWs), and adverse working conditions in Ebola Treatment Units (ETUs).

When treating EVD patients, HCWs must wear personal protective equipment (PPE) to reduce the risk of infection, but wearing PPE in hot and humid conditions can increase the risk of heat strain, reduce performance, and limit the amount of time spent with patients [1,12,13,14,15]. Therefore, we created a simulated ETU to assess HCW performance and safety in delivering critical care while wearing PPE in two different climatic conditions: hot (35 °C, 60% relative humidity) and thermo-neutral (20 °C, 20% relative humidity). In this manuscript, we describe the non-randomized pilot phase of this study, the objective of which was to refine data collection instruments and examine the feasibility of three simulated study tasks important for the care of critically ill EVD patients: peripheral intravenous (PIV) catheter insertion, midline catheter (MLC) insertion, and endotracheal intubation (ETI). This phase was not designed to detect minimally important differences in task performance and participant safety between hot and thermo-neutral conditions.

## 2. Materials and Methods

### 2.1. Study Design

Our ultimate objective was to design a trial that would randomly allocate HCWs to hot vs. thermo-neutral climatic conditions in a simulated ETU and measure performance and safety while in PPE and delivering critical care interventions to simulated patients. Over the course of this non-randomized pilot phase [16] with a small number of study participants, we anticipated the need to modify study procedures. Therefore, we attempted to balance the distribution of professions in hot and thermo-neutral conditions by deliberate (non-random) allocation. We considered a randomized crossover design for the pilot phase but rejected this approach due to concerns that few participants would be willing to spend an entire day in the simulated ETU, even if a sufficiently long washout period between the two simulations could mitigate the learning effect of repeating tasks.

### 2.2. Study Setting

**Figure 1:**
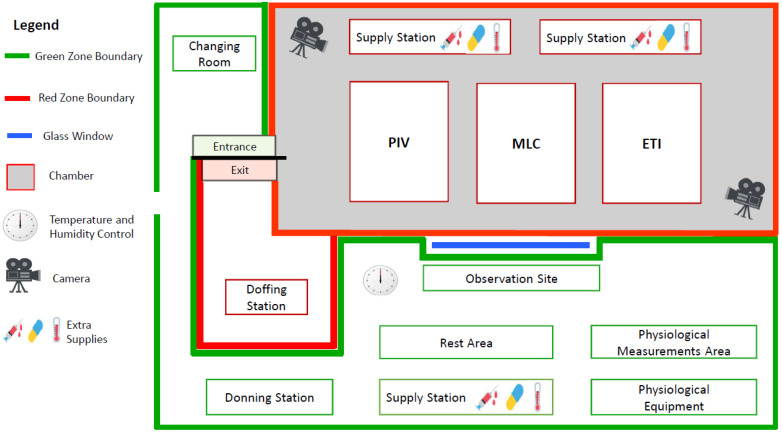
Layout of the simulated Ebola Treatment Unit (ETU). The red zone was divided into the suspect ward (2 beds for suspected Ebola Virus Disease (EVD) patients) and the confirmed ward (1 bed for a confirmed EVD patient), delineated by red and yellow marking tape on the floor. Participants moved unidirectionally from the green zone to red zone, and from the suspected ward (with 2 patients requiring PIV and MLC) to the confirmed ward (with 1 patient requiring ETI) within the red zone. The supply stations in the red zone contained extra supplies for the 3 tasks; additional clean supplies were stored in a green zone supply station. The climate-controlled chamber had only 1 door; in an actual ETU, the entrance to the green zone and the exit from the red zone would be separated. PIV, peripheral IV; MLC, midline catheter; ETI, endotracheal intubation.

All procedures and data collection were done in a simulated ETU at Defence Research and Development Canada (DRDC), Toronto, ON, Canada. A room was designed to resemble the standard layout of an ETU during the 2014–2016 West Africa EVD outbreak [17,18]. The room was partitioned into a ‘green zone’, an area of low infection risk, and a ‘red zone’, an area of high infection risk. The green zone included the PPE-donning area and the investigators’ work area. The red zone included the doffing area and a climate-controlled chamber that housed 3 simulated patients and supply stations (Figure 1). The chamber was set to hot (35 °C, 60% relative humidity) or thermo-neutral (20 °C, 20% relative humidity) climatic conditions.

### 2.3. Study Population

We enrolled hospital-based acute care physicians and nurses who were less than 60 years old, recruiting them by word-of-mouth or via recruitment emails from their institutional or professional groups. Informed consent was obtained. We excluded any HCW with a potential medical condition identified by the self-administered Physical Activity and Readiness Questionnaire (PAR-Q+) [19], corroborated by an interview with the principal investigators, and female HCWs who were pregnant or who considered themselves likely pregnant.

### 2.4. Study Materials

**Figure 2:**
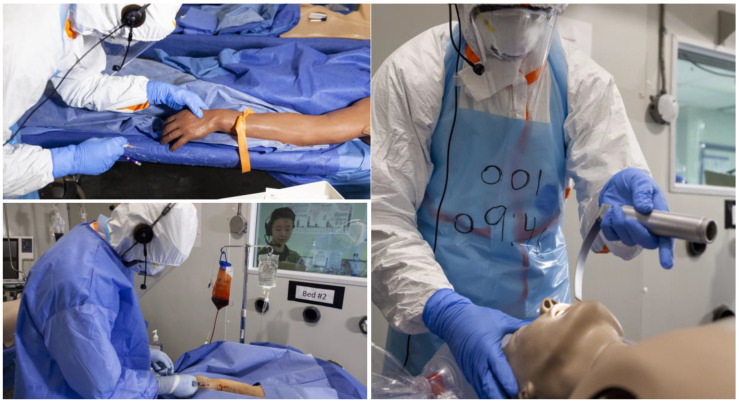
Participant in personal protective equipment (PPE) inserting peripheral intravenous catheter (top left) and midline catheter (bottom left) and performing endotracheal intubation (right).

PPE donning and doffing followed World Health Organization protocols [20]. PPE items included head-to-toe fluid semi-permeable coveralls with a hood and cuffed sleeves (DuPont Tyvek, 800J model TJ198T, Midland, MI, USA); disposable face shields (Critical Coverall, Alpha Pro Tech, Markham, ON, Canada); N95 half-sphere respirators (3M models 1860, 8511 and 8210, Maplewood, MN, USA); disposable plastic aprons; two pairs of long cuffed nitrile gloves; and rubber boots. The PIV catheter, MLC (Bard PowerMidline Catheter, product P4254118, Murray Hill, NJ, USA), and ETI tasks were performed on high fidelity mannequins (Spectrum Nasco, Newmarket, ON, Canada) (Figure 2). Smart Reader Plus 7 data loggers (ACR systems, Surrey, BC, Canada) continuously monitored the temperature and humidity inside the chamber.

### 2.5. Procedures

#### 2.5.1. Participant Orientation

Prior to the study day, participants were emailed a study information package and asked to complete the following tasks: read the consent form, read an informational slide show (Supplementary File S1, complete the medical fitness checklist (PAR Q+) [19] and baseline questionnaire (Supplementary File S2), and watch the 6 instructional videos of the study procedures (http://bit.ly/2kNv1vp (accessed on 31 October 2021)). On the study day, participant orientation included a tour of the chamber and the task equipment, an explanation of the requirements for each procedure (Supplementary File S3) and the safety protocol, and instructions on attaching and operating the physiological equipment.

#### 2.5.2. Participant Monitoring

Heart rate (HR), systolic blood pressure (SBP), and diastolic blood pressure (DBP) were measured with a wireless blood pressure (BP) monitor (Omron Evolv, Model BP7000, Omron Global, Kyoto, Japan), and temperature was measured using a tympanic thermometer (Braun Thermoscan, Braun GmbH, Kronberg, Germany). Participants’ nude weights were recorded (Setra Super Count Remote Scale, Setra, Systems Inc., Boxborough, MA, USA) at baseline and post-simulation to approximate the amount of fluid lost due to sweating, with no intervening oral intake. Participants were fitted with an Equivital EQ02 LifeMonitor vest (model EQ-02-B2-1, Hidalgo Ltd., Cambridge, UK) that recorded HR, respiratory rate (RR), and skin temperature (Figure S1). After donning PPE, participants were given a headset with microphone and speaker to permit two-way communication while in the chamber.

HR, BP, and tympanic temperature were recorded at baseline, after donning PPE (prior to chamber entry), after doffing PPE, and 20–30 min after exiting the chamber. In the chamber, participants’ BP and thermal comfort scale ratings (range, 1–13, with 1 denoting ‘so cold I am helpless’ and 13 denoting ‘so hot I am sick and nauseated’ [21]) were obtained every 10 min. HR, RR, and skin temperature were measured every 15 s and monitored in real time by the study personnel.

Abnormal vital signs were defined as HR exceeding the participant’s age-predicted maximum HR [22], HR < 40 beats/min, and SBP > 180 mmHg or <90 mmHg or a decrease >40 mmHg below baseline. Abnormal vital signs or a thermal comfort scale rating of 12 or 13 would prompt study personnel to conduct a safety check and symptom screen and ask the participant and if they wanted to continue with the simulation.

#### 2.5.3. Chamber and Post-Chamber Procedures

Investigators remained outside the chamber and directly observed participants through a window built into the chamber wall. Investigators began by reading patient case vignettes to participants before the start of each task (Supplementary File S4). Participants completed tasks in order of increasing complexity: PIV catheter insertion, MLC insertion, and ETI (Figure 2). For the PIV and MLC tasks, participants collected blood samples and completed triple packaging according to a modified version of World Health Organization [17,23] and Centers for Disease Control and Prevention [24] guidelines. Participants were video-recorded inside the chamber. To ensure safety, the maximum time in the chamber was 90 min, after which the simulation would be stopped. After completing the tasks, participants exited the chamber and doffed PPE. They were weighed and then offered an electrolyte drink or water to replace lost fluids. Finally, they completed a post-simulation questionnaire (Supplementary File S5).

### 2.6. Outcome Definitions

#### 2.6.1. Task Performance

Tasks included PPE donning and doffing and the 3 procedures (PIV catheter and MLC insertion and ETI). Performance for each task was defined as the proportion of steps completed according to a task-specific score and the time taken for each task. For each task, 2 study personnel independently scored performance on a case report form (CRF, Supplementary File S6-1) using an instruction guide with definitions of grading criteria for each task step (Supplementary File S6-2). We considered completion of >80% of steps for a given task as satisfactory performance. Study personnel also recorded times for task completion. Disagreements were resolved by the 2 observers reviewing the video recording of the simulation session.

#### 2.6.2. Participant Safety

Participant safety was assessed by the number of PPE breaches (both assessor-observed and participant-reported), near-miss incidents, and health-assessment triggers during the simulation. Minor breaches included tears in PPE, touching anywhere around the head or neck, or a fluid splash above the nipple line of the coverall. Major breaches included any needle stick injury, splashes of body fluids onto mucous membranes, and contact of dirty gloves with the inside of PPE, skin, or mucous membranes. Near-miss incidents were noted when a participant recapped a needle, did not immediately dispose of a needle into the sharps bin after completion of task (i.e., placed the needle on a nearby tray), or moved with any sharps or soiled items. Health-assessment triggers were defined by a participant reporting specific symptoms or feeling unwell, abnormal vital signs, or extreme thermal comfort scale ratings (defined above).

#### 2.6.3. Self-Reported Feasibility and Safety

Participants reported feasibility on the post-simulation questionnaire, which included questions on physical and psychological symptoms (e.g., nausea, dizziness, anxiety, frustration); perceived task difficulty and PPE use; and reports of any PPE breaches or near-miss incidents. The effect of each PPE component on the ability of HCWs to provide care was assessed by questions probing interference with the ability to provide care, personal well-being regarding heat, and comfort. Likert [25] responses ranged from 1 (no reduction in the ability to provide care, not hot at all, very comfortable) to 5 (unable to provide care, unbearably hot, very uncomfortable). The average of all responses for each PPE component was calculated.

### 2.7. Data Management and Statistical Analysis

Raw data from paper CRFs were entered into a REDCap database [26]; physiologic data from the Equivital vest were downloaded into a spreadsheet. The sample size was chosen based on availability of time in the climatic chamber and research personnel. We summarized categorical variables as frequencies and proportions and continuous variables as means (standard deviation (SD)) if normally distributed or medians (interquartile range) otherwise. We compared physiological data before, during, and after the simulation and between hot and thermo-neutral conditions. We reported task performance descriptively among physicians and nurses, anticipating differences in prior experience with each task, and compared task performance statistically between hot and thermo-neutral conditions. The purpose of these comparisons was to assess for extreme differences in task performance or safety that would lead to design modifications for the subsequent randomized phase of the study.

We tested for differences between groups using Chi-square or Fisher’s exact tests for categorical variables and Student’s t-test or Wilcoxon rank sum test for continuous variables. Within-subject comparisons used paired t-tests or Wilcoxon signed rank test. All analyses were performed in Excel (version 16.16.3 (181015)) and SAS (version 9.4). Two-sided *p* < 0.05 was interpreted as statistically significant.

## 3. Results

### 3.1. Participant Demographics and Baseline Characteristics

We screened 21 participants (Figure S2), excluded three after medical assessment, and enrolled 18 HCWs in the study. We analyzed results from 18 participants (Table 1).

The average age of participants was 40 (SD 7) years, 50% were female, and most had normal body mass index (<25 kg/m^2^; 11/18). Most were physicians (13/18), worked in critical care (12/18), and had over 10 years working experience (9/16). Only two had previous ETU experience, but 11/18 participants reported work in an austere environment (resource-limited setting, natural disaster, etc.). Most participants reported experience with PIV catheter insertion (14/17) and ETI (14/17), but fewer reported previous experience with MLC insertion (6/17) and triple packaging of highly infectious blood samples (4/15). All participants reported experience with using at least four of six PPE components (gloves, coverall, boots, face shield, face mask, and apron) in their routine work; the majority reported no prior experience using the coverall and face shield. One participant reported a prior history of heat illness. Half the participants reported good or excellent ability to tolerate heat, and half reported an average ability. 

### 3.2. Chamber Settings

Temperature and humidity readings in the chamber varied despite attempts at precise adjustment. The average chamber temperatures were 31.4 (2.3) °C and 20.4 (0.6) °C for the hot and thermo-neutral conditions, respectively, but humidity was inconsistent.

### 3.3. Task Performance

To match participant profession and skill set between the two climatic conditions, we assigned three nurses and five physicians to the hot conditions and two nurses and eight physicians to the thermo-neutral conditions (Figure S2). On average, participants spent 69 (10) min in the chamber (Table 2), with no differences in total chamber time between participants assigned to hot vs. thermo-neutral conditions (*p* = 0.86) or between physicians and nurses (*p* = 1.00). None of the participants spent more than the maximum time of 90 min in the chamber. Participants spent more time on MLC insertion (33 (5) min) than on PIV catheter insertion (16 (6) min) or ETI (16 (8) min) (Table 2).

**Figure 3:**
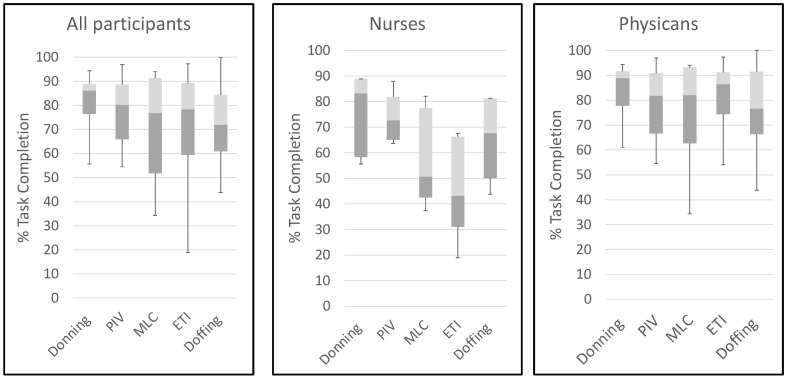
Boxplots showing median percentage (1st and 3rd quartiles) of completed tasks for each procedure for all participants (*n* = 18, left pane), nurses (*n* = 5, middle panel), and physicians (*n* = 13, right panel). ETI and doffing had *n* = 17 (overall) and *n* = 12 (physicians). PIV, peripheral IV; MLC, midline catheter; ETI, endotracheal intubation.

Most participants completed the milestones within each task (Figure 3).

**Figure 4:**
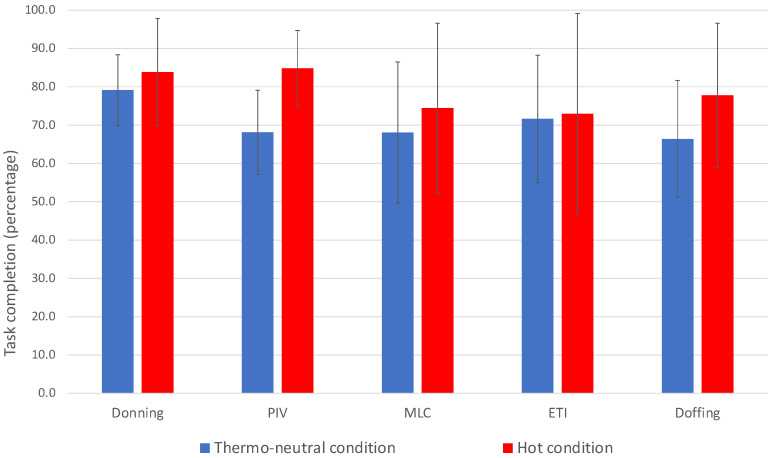
Comparison of the mean percentages of completed tasks between the hot (*n* = 10, red bars) and thermo-neutral (*n* = 8, blue bars) conditions. Data are missing for one participant for ETI and doffing (hot condition). Differences were non-significant for donning (*p* = 0.14), midline catheter (MLC) insertion (*p* = 0.46), endotracheal intubation (ETI) (*p* = 0.60), and doffing (*p* = 0.19) and significant for peripheral IV (PIV) insertion (*p* = 0.015).

Overall, the participants completed 77.4% of steps for PIV catheter insertion, 71.6% for MLC insertion, and 72.3% for ETI. Satisfactory performance (completion of >80% of steps) varied by task: donning (physicians 9/13 (69.2%) vs. nurses 3/5 (60.0%)); PIV catheter insertion (physicians 8/13 (61.5%) vs. nurses 1/5 (20.0%)); MLC insertion (physicians 8/13 (61.5%) vs. nurses 0/5); ETI (physicians 8/13 (61.5%) vs. nurses 0/5); and doffing (physicians 6/13 (46.1%) vs. nurses 2/5 (40.0%)). There were no differences in task completion percentages between the participants assigned to the hot versus thermo-neutral conditions, except for PIV insertion (Figure 4).

### 3.4. Self-Reported Symptoms and Concerns

There were 31 symptoms and concerns (median per participant, 3.0 (2.0–4.0)) from the participants in the hot condition compared to 17 (median per participant, 1.5 (1.0–2.8)) in the thermo-neutral condition (*p* = 0.28) (Figure S3).

Sixteen of 18 participants reported at least one symptom or concern, with the three most common being frustration at not doing the tasks well, inability to focus on tasks or think clearly, and feeling uncomfortably hot (Figure S3). None of the participants in either condition reported muscle aches, numbness, or tingling while in the chamber.

Participants reported similar ease of use for each PPE component and negative effects on the ability to provide care in hot and thermo-neutral conditions (Figure S4).

### 3.5. Participant Safety

There were eight health-assessment triggers in five participants, of whom four had been assigned to hot conditions (Table 3). All the health-assessment triggers (*n* = 8) happened during ETI or at the end of the MLC task, i.e., towards the end of the individual participant simulation. Two participants had SBP > 180 mmHg, four participants had extreme discomfort on the thermal comfort scale, one participant felt pre-syncopal, and one participant reported feeling nauseous and unwell. We stopped the simulation for two participants, both in the hot conditions, who reported a thermal comfort score of 12. The remaining three participants elected to continue with the simulation, of whom two had thermal comfort scores of 12 and 13.

There were 47 breaches (median per participant, 2.0 (1.0–4.0)), most of which happened during the doffing phase (31/47, 66.0%). All breaches were minor and included tears in PPE (*n* = 3), attempting to adjust facial PPE (*n* = 3), fluid splashes on PPE or intact skin (*n* = 13), and actual doffing errors (*n* = 28). No participant had a major breach. Sixteen participants had at least one minor breach; one participant had 10 breaches and two participants had five breaches each. There were 44 near-miss incidents (median per participant, 2.0 (1.0–4.0)), with the majority occurring during MLC insertion (23/44, 52.3%). Near miss-incidents involved recapping needles, walking with sharps, walking with soiled items, and tripping.

**Figure 5:**
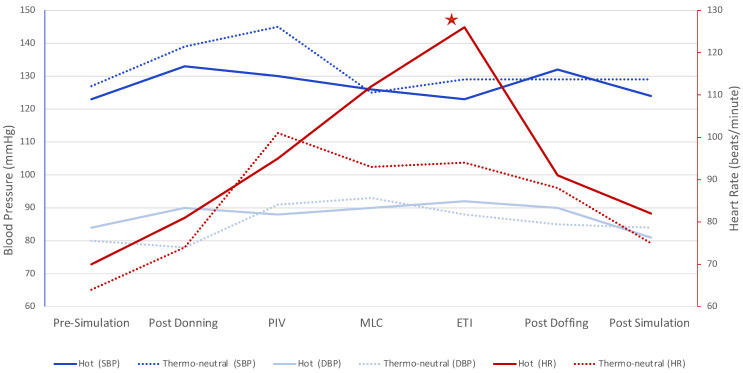
Changes in medians of systolic blood pressure (SBP), diastolic blood pressure (DBP), and heart rate (HR) in both hot (solid lines) and thermo-neutral (dotted lines) conditions at seven time points throughout the simulation. Tasks included peripheral intravenous catheter insertion (PIV), midline catheter insertion (MLC), and endotracheal intubation (ETI). The only significant difference in vital signs between hot and thermo-neutral conditions was for HR during endotracheal intubation, denoted by the star (*p* = 0.029).

Overall, median SBP and median DBP increased from baseline values (SBP 124 mmHg, DBP 81 mmHg), peaked during procedures (SBP 138 mmHg, DBP 88 mmHg during PIV catheter insertion), and then returned to baseline values (SBP 124 mmHg, DBP 81 mmHg) post-simulation (*p* = 0.31 and *p* = 0.51 for pre-post differences in SBP and DBP, respectively; Figure 5). The median baseline HR was 66 bpm, which peaked during ETI (118 bpm) and then returned to 79 bpm at post-simulation (*p* = 0.0005 for pre-post difference in HR; Figure 5).

Participant skin temperature was higher in hot conditions (median (range) 36.7 (35.4–37.4) °C) than in thermo-neutral conditions (35.4 (32.8–37.0) °C; *p* = 0.051). Tympanic temperature increased in hot conditions after the simulation vs. baseline (median (range) 0.45 (0.00–0.80)) but was stable in thermo-neutral conditions (0.10 (−0.20 to +0.90 °C); *p* = 0.14 for difference between hot and thermo-neutral conditions). Participants’ median (range) weight loss was −0.55 (−0.90 to 0.10) kg in hot conditions and −0.20 (−0.50–0.00) kg in thermo-neutral conditions (*p* = 0.042 for difference between hot and thermo-neutral conditions).

### 3.6. Protocol Changes

The major protocol change was to align care tasks with previous skills and experience. Given the inpatient or emergency medicine clinical background of physician participants, we did not change their tasks. Based on nurses’ feedback regarding lack of experience with MLC insertion and ETI in their usual practice, we revised their tasks to include insertion of a nasogastric tube, insertion of a urinary catheter, setting up of an infusion pump for selected medications, and setting up a continuous renal replacement therapy machine. These tasks are aligned with nursing expertise and with current and anticipated future best practices for critically ill patients in EVD outbreaks. Another important change was the completion of repairs to the climatic chamber to enable reliable temperature and humidity control. Detailed protocol changes, which will be implemented and assessed in the randomized phase of the study, are listed in Table 4.

## 4. Discussion

We designed this non-randomized pilot study to assess the feasibility of measuring HCW performance and safety as HCWs delivered critical care interventions in a simulated ETU under ‘hot’ (35 °C, 60% relative humidity) and ‘thermo-neutral’ (20 °C, 20% relative humidity) conditions. We designed a simulated ETU in accordance with established guidance [17] and standardized and calibrated study procedures and data collection methods. Numerically, more physicians than nurses achieved satisfactory completion of each task (PPE donning and doffing, PIV catheter and MLC insertions, ETI), likely related to differences in scope of professional practice and experience in austere environments, and possibly related to a smaller group of nurses participating in the study. Nonetheless, this finding led us to modify tasks to align with acute care nursing expertise for the next phase of the study. Two of 18 participants in the hot conditions terminated the simulation because of symptoms of thermal discomfort, and there were no major safety breaches. In all participants completing the simulation, vital signs returned to baseline 20–30 min after exiting the chamber.

Similar to our findings, a study of 25 HCWs acclimatized to the hot and humid climate in West Africa found that they worked an average of 65.7 min per session in the ETU (mean temperature 29.6 °C, humidity 65.4%) and reported moderate but safe thermal strain [13]. Acclimatization to local weather conditions is helpful for HCWs before deployment to outbreaks in hot regions [27]. Given our short study duration, non-tropical location, and non-outbreak setting, it was impractical to acclimatize participants to hot conditions over many days to weeks. Since 16 of 18 participants were able to complete all tasks, it may be feasible to rapidly assemble and train teams to provide critical care to patients without lengthy acclimatization periods.

The use of semi-impermeable PPE presents an impaired heat evaporative environment for HCWs and, along with long periods of clinical activity, may induce heat illness that impairs cognition and task performance [15,27,28,29]. We found that most breaches and health-assessment triggers occurred towards the end of the simulation, when both task complexity and elapsed chamber time had increased, and seven of eight health-assessment triggers occurred in participants assigned to the hot climatic condition. In the next phase of this study, we plan on measuring baseline and post-simulation measures of cognitive performance to assess whether PPE-related heat strain impairs cognitive function.

This non-randomized pilot study has limitations. First, we did not randomly assign participants to hot vs. thermo-neutral conditions, and our sample size was insufficient to assess minimally important differences in task performance and safety. Instead, our objective was to refine study procedures. Second, it is possible that having participants watch procedural videos and read materials regarding study tasks (‘passive learning’) rather than in-person training with the opportunity to practice tasks (‘active learning’) may have reduced task performance. A study of emergency medicine residents compared video-based learning to traditional lectures for disaster medicine and found no significant differences in knowledge or comfort scores, but the video-based learning group had better task performance [30]. Third, HCW performance in a simulated ETU may be different from a real outbreak. However, simulation has the benefits of standardizing procedures on mannequins, with less risk to HCWs and zero risk to patients. In this study, only two of 18 participants had prior ETU experience, precluding analyses of the effect of experience on task performance and safety. Fourth, the semi-permeable PPE in this study, even with the additional Equivital vest, is more breathable than the impermeable PPE used by some NGOs, which may attenuate differences in task performance and safety between hot vs. thermo-neutral climatic conditions. Fifth, the chamber climate controls functioned imperfectly, with variable humidity and a temperature separation between hot and thermo-neutral conditions of 11 °C rather than the planned 15 °C.

Limited data support the performance and safety of HCWs providing critical care to EVD patients while in PPE and in field conditions [8,9,10,12,13,14]. This pilot simulation study provides a model for studying care provision to patients with highly infectious pathogens, including EVD, Lassa fever, and Crimean-Congo hemorrhagic fever, and could also be used to study healthcare procedures specific to critical illness syndromes caused by these pathogens, variations in PPE, and other aspects of working conditions. This model might provide a platform for medical organizations, institutions, and HCWs to practice procedures and improve pre-deployment preparedness planning and infection prevention and control. In the next phase of the study, we will randomize participants to hot or thermo-neutral conditions and measure task performance and participant safety.

## 5. Conclusions

This non-randomized pilot study, providing critical care in a simulated ETU, found that participation appeared safe, with two participants developing symptoms of thermal discomfort. Task feasibility was generally higher for physicians, likely related to the usual scope of practice. Data collection tools and study procedures were iteratively refined and tested for the subsequent phase of the study.

## Figures and Tables

**Table 1:** Participant demographics and baseline characteristics.

Characteristic	*n* = 18
**Age,** years, mean (SD)	40 (7)
**Body mass index,** kg/m^2^, mean (SD), *n* (%)	24.5 (5.8)
<25	11 (61.1)
25–29.9	6 (33.3)
≥30	1 (5.6)
**Sex,** Female, *n* (%)	9 (50)
**Profession,***n* (%)	
Nurse	5 (27.8)
Physician	13 (72.2)
**Clinical specialty,***n* (%)	
Critical Care	12 (66.6)
Emergency Medicine	3 (16.7)
Other in-patient care	3 (16.7)
**Work experience,** years, *n* (%) (*n* = 16)	
<10	7 (43.8)
10–20	6 (37.4)
>20	3 (18.8)
Work experience in an ETU, *n* (%)	2 (11.1)
Work experience in austere environments, *n* (%)	11 (61.1)
**Prior procedural experience,***n* (%)	
Peripheral intravenous catheter insertion (*n* = 17)	14 (82.4)
Midline catheter insertion (*n* = 17)	6 (35.3)
Central venous catheter insertion (*n* = 17)	14 (82.4)
Endotracheal intubation (*n* = 17)	14 (82.4)
Triple packaging of blood samples (*n* = 15)	4 (26.7)

ETU, Ebola Treatment Unit; SD, standard deviation.

**Table 2:** Participant task completion times ^1^.

	Total Time in Chamber (*n* = 18)	PIV Catheter (*n* = 18)	Midline Catheter (*n* = 17)	Endotracheal Intubation (*n* = 17)
All	68.5 (10.3)	15.7 (5.7)	33.3 (4.9)	15.6 (7.9)
Hot conditions (*n* = 10)	69.7 (9.5)	18.0 (4.9)	35.2 (3.3)	14.0 (5.3)
Thermo-neutral conditions (*n* = 8)	67.0 (11.9)	12.9 (5.8)	31.3 (5.7)	17.4 (10.2)
Nurses (*n* = 5)	65.6 (8.3)	13.9 (3.9)	33.4 (4.4)	13.1 (2.2)
Physicians (*n* = 13)	69.6 (11.1)	16.5 (6.3)	33.3 (5.3)	16.6 (9.2)

^1^ All times are in minutes (mean (SD)).

**Table 3:** Health-assessment triggers, minor breaches, and near-miss incidents.

Type of Event	Condition	No. of Events (Participants)	Donning, *n*	Doffing, *n*	PIV, *n*	MLC, *n*	ETI, *n*
Health-Assessment Trigger	Hot	7 (4)	0	0	0	4	3
	Thermo-neutral	1 (1)	0	0	0	0	1
Minor Breach	Hot	26 (9)	1	14	2	5	2
	Thermo-neutral	21 (7)	0	17	1	1	2
Near-Miss Incident	Hot	21 (7)	0	0	8	11	2
	Thermo-neutral	23 (8)	0	0	11	1	0

ETI, endotracheal intubation; MLC, midline catheter; PIV, peripheral intravenous catheter.

**Table 4:** Summary of protocol changes based on pilot study.

Item	Study Changes	Rationale
Procedural tasks	Changes to the nurses’ procedural tasks	To align tasks with the expertise of acute care nurses, new tasks were developed (nasogastric tube insertion, urinary catheter insertion, administration of drugs via infusion pumps, and set-up of a CRRT machine).
Randomization	Stratified randomization	In the subsequent trial, randomization will be stratified by professional status (nurse vs. physician).
Cognitive tasks	Addition of objective assessment of stress effects	To objectively measure how heat stress affects HCWs’ cognitive function, we added tests of cognitive performance to complement subjective measures (i.e., post simulation questionnaire). Computer-based cognitive tasks will assess attention, decision-making, reaction time, learning, spatial memory, and working memory before and after the simulation.
Data collection tools	Addition of Instructor Guide	The Instructor Guide provides detailed instructions on CRF completion and defines grading criteria for each task item, thus reducing inter-rater variability.
	Refinement of data tools	We eliminated redundant items from the CRF and questionnaires.
	Addition of video cameras	Multiple video cameras record the simulation and help data collectors to be accurate.
Equipment	Equivital© for temperature measurement	The previous four skin thermistors to measure temperature required shaving participants and complex data computation. The simpler Equivital© device is now used.
	Ihealth© to Omron© BP cuff	The Omron© BP cuff can function without Bluetooth while in the chamber, unlike Ihealth©.
	Chamber repairs	To ensure precise control of the temperature and humidity inside the climatic chamber.
Monitoring participant safety	Eliminated pre-doffing vitals	Removal of pre-doffing vitals improved procedure flow and reduced task interruption.

BP, blood pressure; CRF, case report form; CRRT, continuous renal replacement therapy; HCW, healthcare worker.

## Data Availability

The data presented in this study are available on reasonable request from the corresponding authors.

## Supplementary Materials

The following is available online at https://www.mdpi.com/article/10.3390/v13112205/s1 (all in one file), Figure S1: Physio-logical equipment used in the pilot study. The Equivital vest measured heart rate (HR), respira-tory rate, and skin temperature. The wireless Omron BP cuff measured HR and systolic and di-astolic blood pressure. Separate skin thermistors were taped to the chest, upper arm, upper thigh, and shin; they measured skin temperature throughout the simulation; Figure S2: Flow of participants through the study; Figure S3: The percentages of participants in the hot condition (red bars) and thermo-neutral condition (blue bars) that reported health symptoms and concerns in the post-simulation questionnaire; Figure S4: Ease of use of different PPE components while delivering care in both hot (red bars) and thermo-neutral (blue bars) conditions. Scores ranged from 0 to 5, with a higher score denoting increased difficulty to deliver care as a result of the PPE component. Supplementary Files S1–S6 are also contained in this file. Supplementary File S1: In-formational Slide Show; Supplementary File S2: Baseline Questionnaire; Supplementary File S3: Simulated ETU Orientation Script; Supplementary File S4: Patient Vignettes; Supplementary File S5: Post-Simulation Questionnaire; Supplementary File S6-1. Case Report Form; Supplementary File S6-2. Instruction Guide for Case Report Form.
