# Supplementary material for: Establishing Healthcare Worker Performance and Safety in Providing Critical Care for Patients in a Simulated Ebola Treatment Unit: Non-Randomized Pilot Study
Source: Viruses. 2021 Nov 2;13(11):2205. doi: 10.3390/v13112205 (PMC8622862; doi:10.3390/v13112205)
Supplement: Supplementary file 1 [file viruses-13-02205-s001.zip › viruses-1335932-Supplementary.pdf]

Establishing Healthcare Worker Performance and Safety in Providing Critical Care for Patients in a Simulated Ebola Treatment Unit: Pilot Study

Peter Kiiza *et al*

Supplementary Material

Supplementary Figures

Legends for Supplementary Figures

- Figure S1: Physiological equipment used in the pilot study. The Equivital vest measured heart rate (HR), respiratory rate, and skin temperature. The wireless Omron BP cuff measured HR and systolic and diastolic blood pressure. Separate skin thermistors were taped to the chest, upper arm, upper thigh, and shin; they measured skin temperature throughout the simulation.
- Figure S2: Flow of participants through the study
- Figure S3: The percentages of participants in the hot condition (red bars) and thermo-neutral condition (blue bars) that reported health symptoms and concerns in the post-simulation questionnaire. For symptoms without a bar of a particular colour, the percentage is zero.
- Figure S4: Ease of use of different PPE components while delivering care in both hot (red bars) and thermo-neutral (blue bars) conditions. Scores ranged from 0 to 5, with a higher score denoting increased difficulty to deliver care as a result of the PPE component.

Figure S1

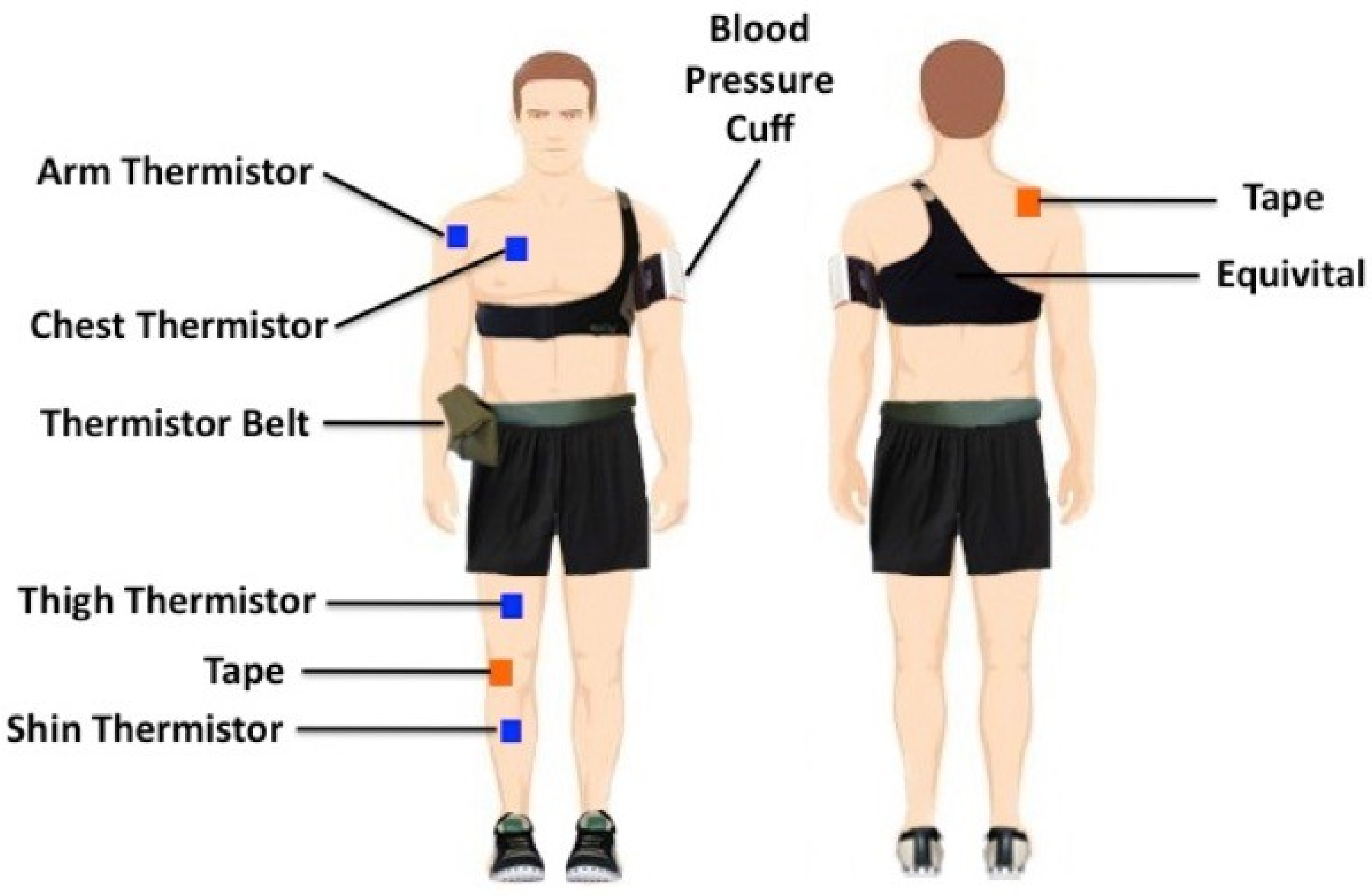
Blood Pressure Cuff
Arm Thermistor
Chest Thermistor
Thermistor Belt
Thigh Thermistor
Tape
Shin Thermistor
Tape
Equivital

Figure S2

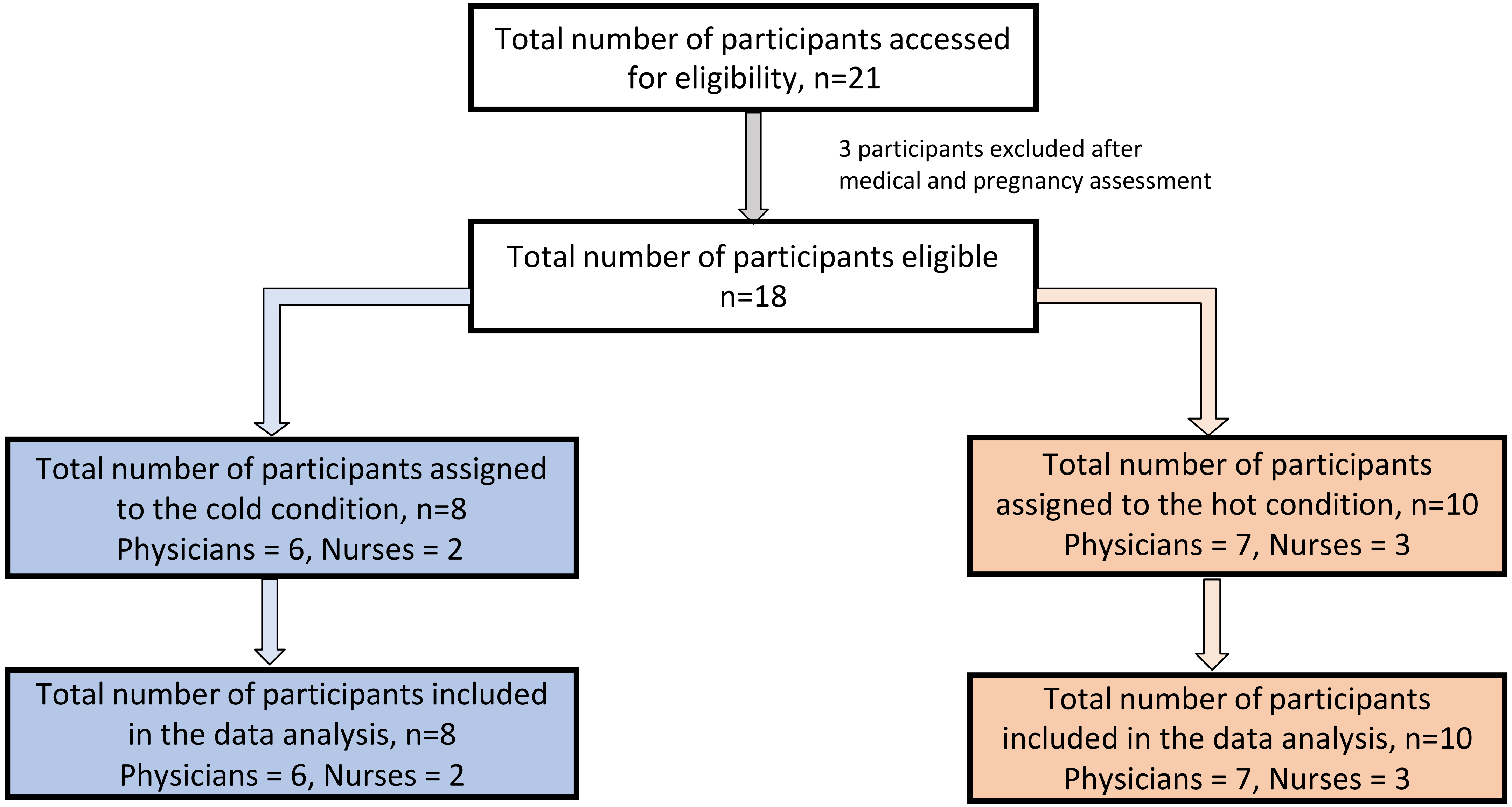
Total number of participants accessed for eligibility, n=21
3 participants excluded after medical and pregnancy assessment
Total number of participants eligible n=18
Total number of participants assigned to the cold condition, n=8
Physicians = 6, Nurses = 2
Total number of participants assigned to the hot condition, n=10
Physicians = 7, Nurses = 3
Total number of participants included in the data analysis, n=8
Physicians = 6, Nurses = 2
Total number of participants included in the data analysis, n=10
Physicians = 7, Nurses = 3

Figure S3

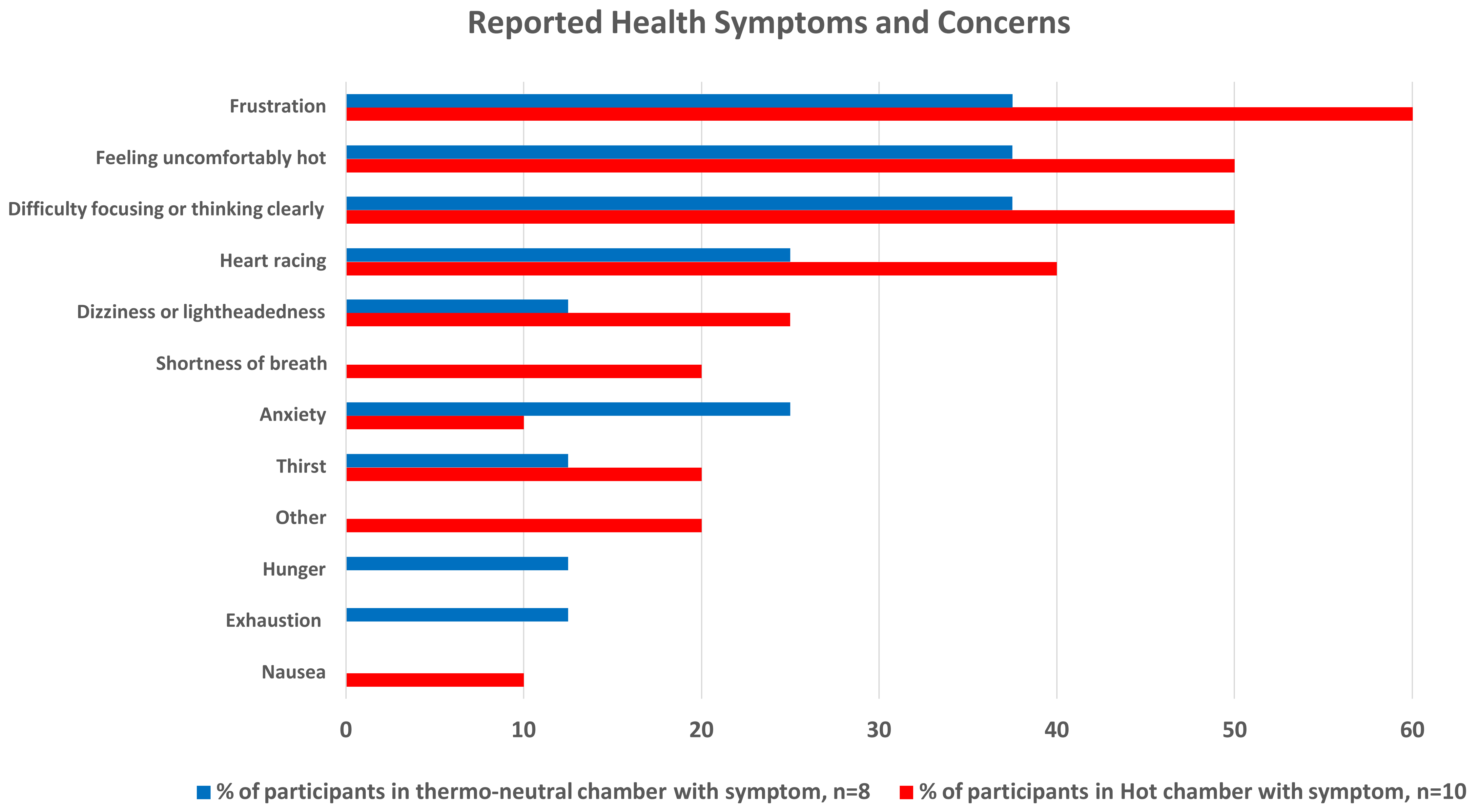
Reported Health Symptoms and Concerns
Frustration
Feeling uncomfortably hot
Difficulty focusing or thinking clearly
Heart racing
Dizziness or lightheadedness
Shortness of breath
Anxiety
Thirst
Other
Hunger
Exhaustion
Nausea
0
10
20
30
40
50
60
% of participants in thermo-neutral chamber with symptom, n=8
% of participants in Hot chamber with symptom, n=10

Figure S4

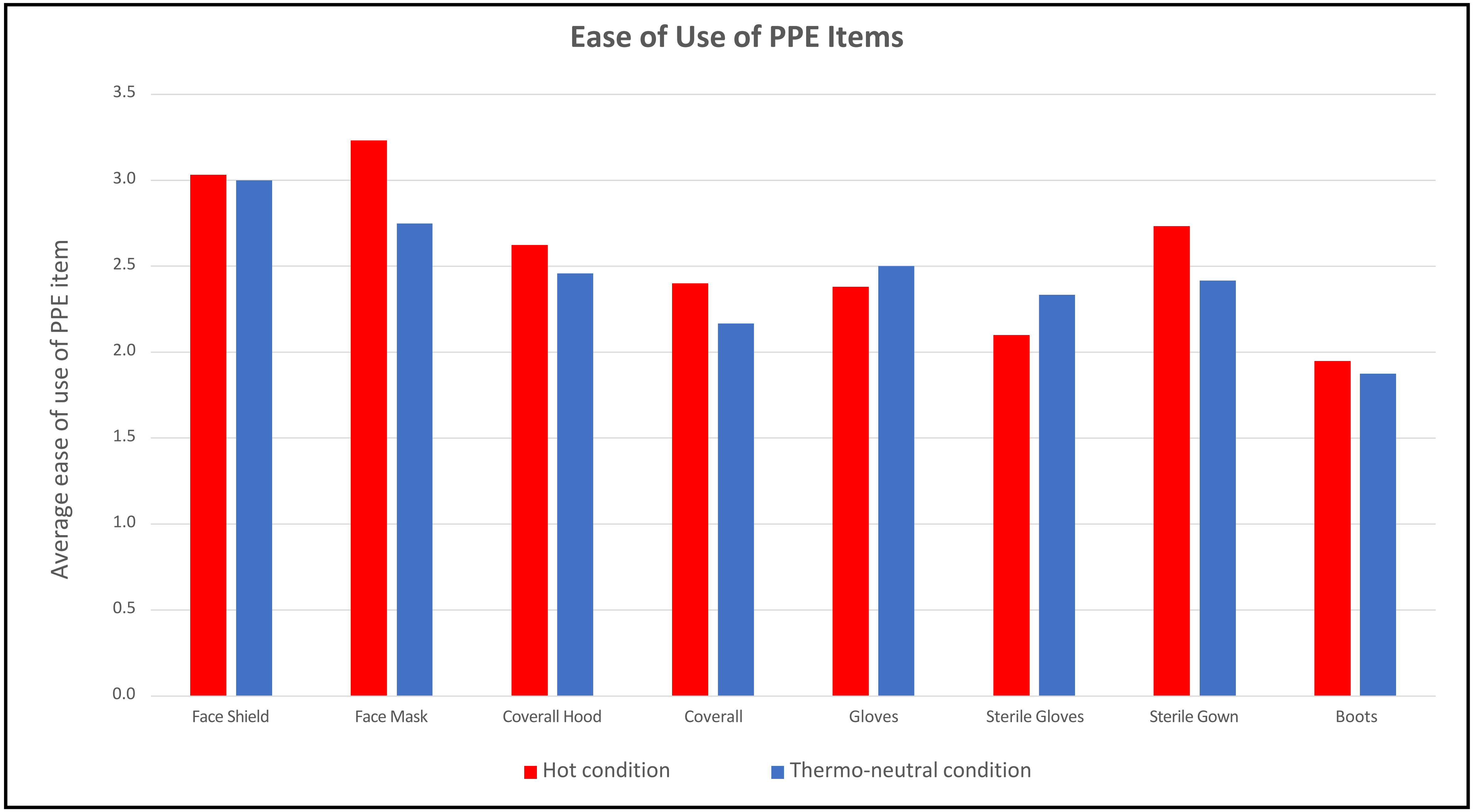

Ease of Use of PPE Items
Average ease of use of PPE item
3.5
3.0
2.5
2.0
1.5
1.0
0.5
0.0
Face Shield
Face Mask
Coverall Hood
Coverall
Gloves
Sterile Gloves
Sterile Gown
Boots
Hot condition
Thermo-neutral condition

File S1: Study information slides

Ebola Study Information

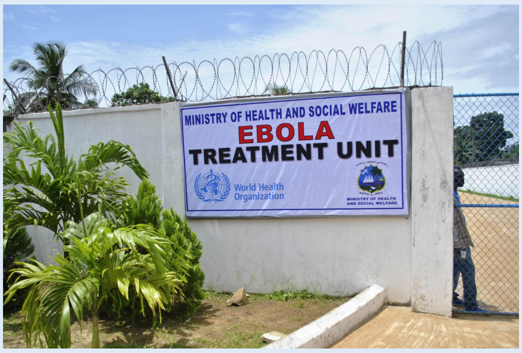

Establishing Feasibility & Safety of Providing Critical Care for Patients with Ebola through design of a simulated Ebola Treatment Centre

1

Before the Study

Please ensure that you:

1. Book your study date here: https://ebolasimulationcalender.youcanbook.me/

2. Fill out the Pre-Simulation Questionnaire and PAR-Q here: https://redcap.sunnybrook.ca/redcap/surveys/?s=DR7RH8LCJJ

* Please complete both surveys ***at least 3 days*** prior to the study (as soon as possible is preferred)*

3. Watch the 6 videos of the simulation procedures: https://www.youtube.com/watch?v=bgcfH_MuuW0&list=PLwYJP97SSV14EVYeqd5I94elLTh3RQsp7

4. Read the Volunteer Consent Form

2

What to Bring

1. Loose fitting shorts
2. A change of clothes for after the simulation
3. Scrubs (*optional - if you do not have your own scrubs we have fresh scrubs available in various sizes)

What to Wear

- Comfortable undergarments
- Comfortable bra (ladies)
- Socks
- Comfortable footwear

(i.e. items you would usually wear with your scrubs)

3

Brief Overview of Study Day

Total Study Time: 2- 4 hours

- Orientation and tour of facility (15-20 min)
- Baseline vitals (5-10min)
- Changing and attaching physiological equipment (15-20 min)
- Donning PPE (10-15min)
- 3 tasks in simulation chamber (60-90 min)
- Doffing PPE (10-15min)
- Changing and removing physiological equipment (10-15 min)
- Post-simulation questionnaire, debriefing, and snacks (15-20 min)

4

Directions

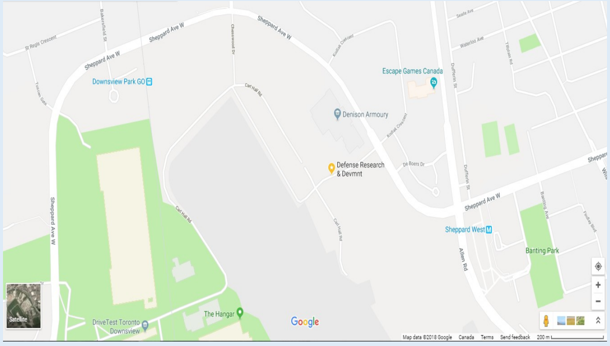

Address: 1133 Sheppard Avenue West
North York, ON
Canada
M3K 2C9

Entrance and parking are only accessible from Yukon Lane

Driving and Parking instructions:

1. Copy the DRDC address into google maps: www.google.ca/maps
2. Click directions and enter your location
3. After turning on to Yukon Lane, the DRDC is the BLACK building on your LEFT (East side of Yukon Lane)
4. Visitor parking is located in front

Transit instructions:

- Take the TTC to Sheppard West (formerly Downsview) station and exit
- Walk approximately 500m west on Sheppard Ave. W
- Turn LEFT (South) on Yukon Lane
- The DRDC is the BLACK building on your LEFT (East side of Yukon Lane)

(Alternatively: 84, 106, 108 westbound buses will depart from Sheppard West and stop on Sheppard Ave. W at Kodiak Crescent Avenue East)

5

Directions (cont'd)

- After you arrive, please proceed to the gatehouse located adjacent to the visitor parking lot.
- The security staff will notify the study team of your arrival, and a study team member will come meet you at the gatehouse to escort you inside the study facility.
- Should you require assistance on the day of study, please feel free to text or call:
  - Sarah at (416) 931-0804 OR Peter at (437) 996-1136

6

Equipment

These pieces of equipment will be used to measure your vitals during the simulation

- Skin Thermistors
- Equivital
- Blood Pressure Cuff

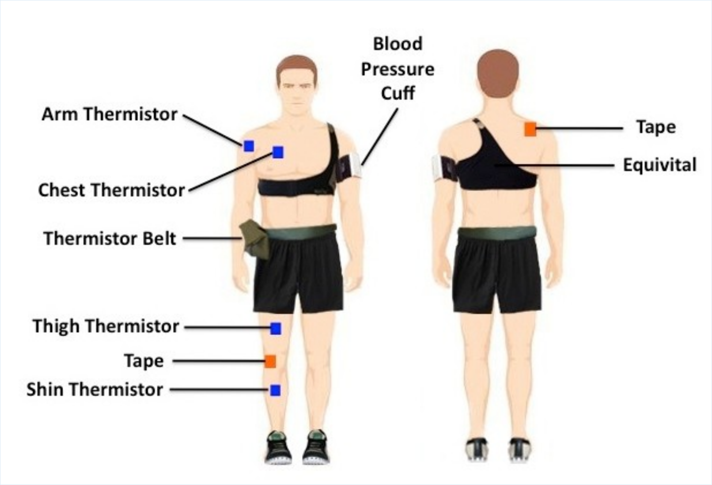

7

Skin Thermistors

- Measures and records skin temperature
- Will be attached using medical tape to the arm, chest, thigh and shin

Please advise the study team if you are allergic to adhesives

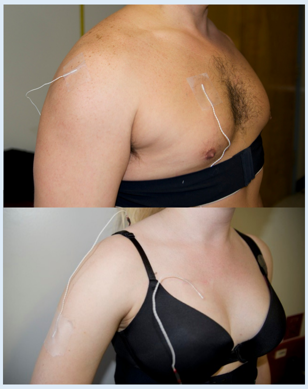

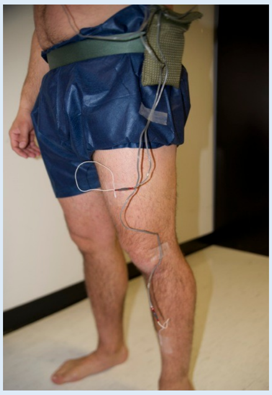

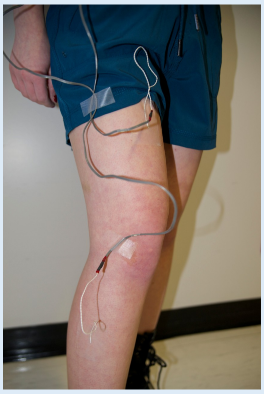

8

Equivital

- Measures and records heart rate and respiratory rate

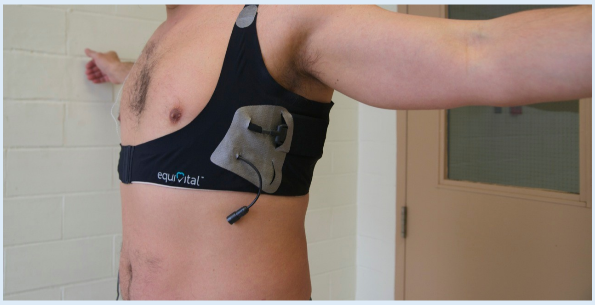

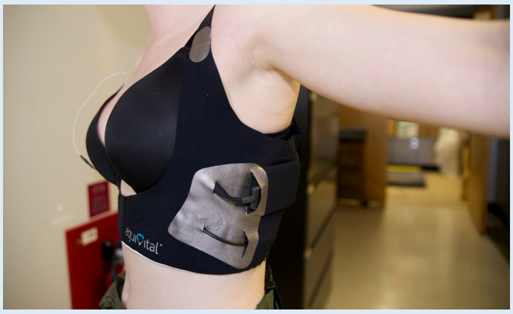

Blood Pressure Cuff

- Measures blood pressure during the simulation
- The cuff will be placed on non-dominant arm
- We will monitor your blood pressure during the simulation via a wireless bluetooth application
- Every 10 minutes you will be asked to pause for a reading. At this time try to keep your arm as still as possible

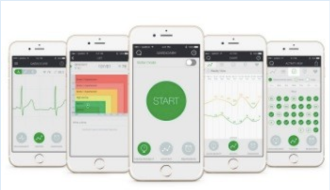

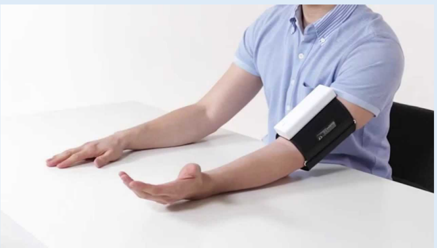

Additional Points

- Change rooms will be available for you to change in and out of scrubs.
- We will need to take your weight before and after the simulation as an approximate measure of body water loss. For accuracy, please ensure you remove ALL clothing before stepping on the scale. To protect your privacy, the scale will be located inside the change room
- After the simulation, showers, towels, and toiletries will also be available.
- Participants will be given 67$ and reimbursed for parking and travel-related expenses.

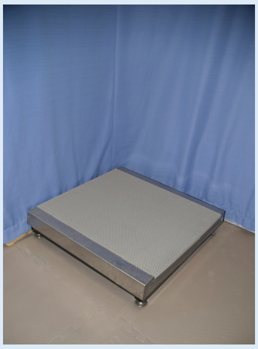

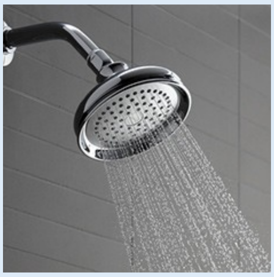

11

Questions?

- If you have any questions or concerns, please do not hesitate to contact the study team.

ebolasimulation@sri.utoronto.ca

Thank you for your participation!

12

File S2: Pre-Simulation Questionnaire

https://redcap.sunnybrook.ca/redcap/surveys/?s=DR7RH8LCJJ

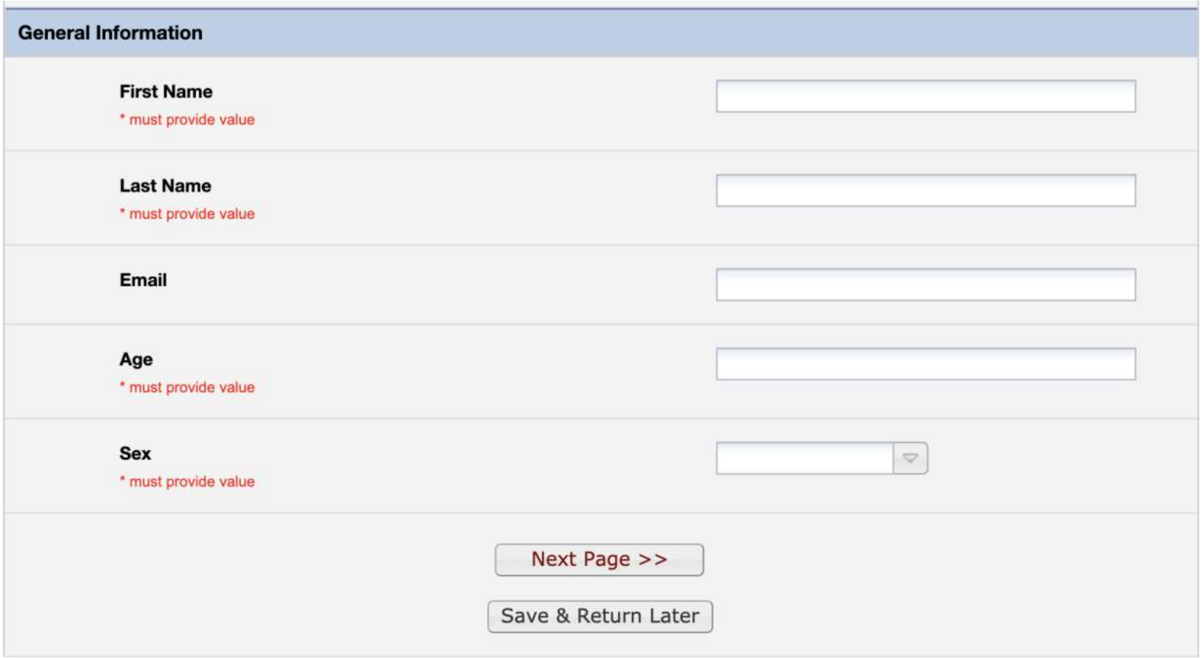

General Information

First Name
* must provide value

Last Name
* must provide value

Email

Age
* must provide value

Sex
* must provide value

Next Page >>

Save & Return Later

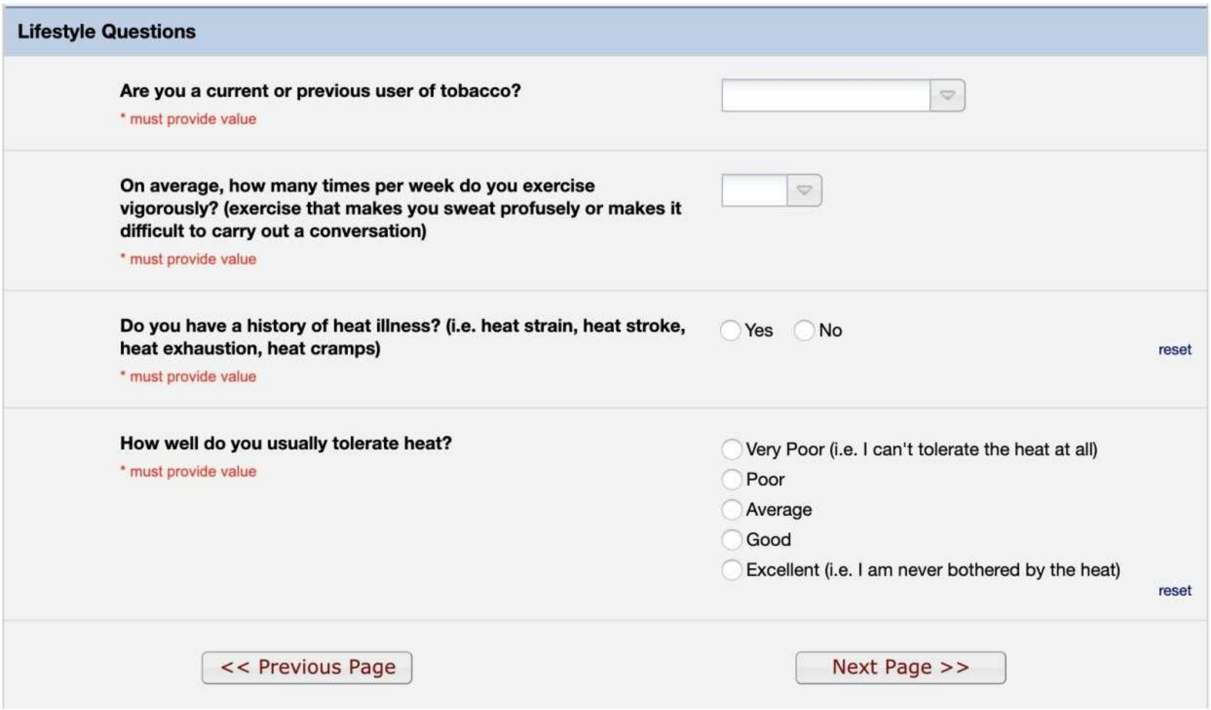

Lifestyle Questions

Are you a current or previous user of tobacco?
* must provide value

On average, how many times per week do you exercise vigorously? (exercise that makes you sweat profusely or makes it difficult to carry out a conversation)
* must provide value

Do you have a history of heat illness? (i.e. heat strain, heat stroke, heat exhaustion, heat cramps)
* must provide value

Yes No

reset

How well do you usually tolerate heat?
* must provide value

Very Poor (i.e. I can't tolerate the heat at all)
Poor
Average
Good
Excellent (i.e. I am never bothered by the heat)

reset

<< Previous Page

Next Page >>

Current Occupation

What is your current occupation?
* must provide value

How many years have you worked in your current occupation?
* must provide value

In which country did you complete the majority of your medical training?
* must provide value

What department do you work in?
* must provide value

<< Previous Page   Next Page >>

Save & Return Later

How often do you typically perform the following procedures in your work?

	Never	Rarely (i.e. once a year)	Sometimes (i.e. once a month)	Often (i.e. once a week)	Frequently (i.e. once a day)	
**Peripheral IV Cannula Insertion** * must provide value	○	○	○	○	○	reset
**Mid-line Catheter Insertion** * must provide value	○	○	○	○	○	reset
**Central Venous Catheter (CVC) Insertion** * must provide value	○	○	○	○	○	reset
**Peripherally Inserted Central Catheter (PICC) Insertion** * must provide value	○	○	○	○	○	reset
**Intubation** * must provide value	○	○	○	○	○	reset
**Mechanical Ventilation** * must provide value	○	○	○	○	○	reset
**Triple Packaging of Laboratory Samples** * must provide value	○	○	○	○	○	reset

<< Previous Page   Next Page >>

Save & Return Later

How often do you typically don/doff the following PPE items in your work?

	Never	Rarely (i.e. once a year)	Sometimes (i.e. once a month)	Often (i.e. once a week)	Frequently (i.e. once a day)	
**Non-Sterile Gloves** * must provide value	○	○	○	○	○	reset
**Sterile Gloves** * must provide value	○	○	○	○	○	reset
**Face Mask** * must provide value	○	○	○	○	○	reset
**Face Shield** * must provide value	○	○	○	○	○	reset
**Coverall** * must provide value	○	○	○	○	○	reset
**Sterile Gown** * must provide value	○	○	○	○	○	reset
**Rubber Boots** * must provide value	○	○	○	○	○	reset

<< Previous Page   Next Page >>

Save & Return Later

Previous Experience

Do you have experience providing health care in austere environments? (Check all that apply)
* must provide value

- ☐ Natural Disaster
- ☐ Armed Conflict
- ☐ Refugee or Internally Displaced Persons Camp
- ☐ Resource Limited Setting (abroad)
- ☐ Other
- ☐ I do not have experience working in austere environments

Do you have experience working in an Ebola Treatment Unit?
* must provide value

○ Yes ○ No   reset

Do you have experience working in any other outbreak situations? (ie. SARS outbreak)

* must provide value

○ Yes ○ No   reset

<< Previous Page   Next Page >>

Save & Return Later

Simulation Questions

**Scrub Size**<br>* must provide value	[dropdown]
**Glove Size**<br>* must provide value	[dropdown]
**Sterile Glove Size**<br>* must provide value	[dropdown]
**Shoe Size**<br>* must provide value	[dropdown]
**What is your approximate height?**<br>* must provide value	(cm)

<< Previous Page   Next Page >>

Save & Return Later

How challenging do you expect each task that you will be performing in the study to be?

	Very Easy	Easy	Neither easy nor difficult	Difficult	Very Difficult	
**Donning PPE**<br>* must provide value						rese
**Doffing PPE**<br>* must provide value						rese
**IV Insertion**<br>* must provide value						rese
**Mid-line Catheter Insertion**<br>* must provide value						rese
**Intubation**<br>* must provide value						rese
**Triple Packaging of Laboratory Samples**<br>* must provide value						rese

<< Previous Page   Submit

Save & Return Later

File S3: Simulated ETU Orientation Script

Note: The text in green color represents key aspects that require the study personnel to demonstrate to the participant, record physiologic measurements and/or time recordings, and/or offer detailed explanations to the participant.

INTRODUCTION

Welcome to our study. Thank you for taking the time out of your busy day to come here. Here are the study team members that will be with us today. **<introduce team>** (allow the participant to put any bags or jackets in the changeroom).

So, today we are first going to give you a quick tour around the facilities and show you the equipment we will be using for the simulation.

The whole study will take anywhere between 2-4 hours, so we recommend using the restroom facilities at some point before putting on the PPE. If you need to use the restrooms let us know and one of us can escort you there.

Would you like grab any snacks or beverages before we begin the tour?

DONNING/DOFFING STATION

So this is the donning and doffing area.

The red and green lines delineate the red and green zones. The red zone is where PPE must be worn because there is a higher risk of infection. The green zone is where there is a much lower risk, so PPE does NOT have to be worn. Once you enter the red zone you cannot return to the green zone until you doff your PPE.

This is the **donning** station. This is where you will put on your PPE before entering the simulation room. Here is your rubber boots, coverall, inner and outer gloves, sanitizer, apron, face mask and face shield. We recommend using this anti-fog spray for your face shield. If you have long hair, don't forget to tie your hair back and put on a hair net BEFORE you put on your facemask.

If you are having trouble remembering the steps, there are posters here on the wall to guide you.

This is the PPE **doffing** area. These 2 bins are for disinfecting your rubber boots. One is for when you exit the chamber and the other for when you enter the green zone.
You can put your patient blood samples in this sealable transport bin after you come out of the chamber. While doffing, it is really important to work slow and take your time because it is when have the highest risk of getting infected.

Vitals

We will be taking your vitals at 5 different points during the study. Twice before you enter the chamber and 3 times after you exit. The first is a baseline measurement. The second measurement is done right before you enter the chamber and the third measurement is done

right after you exit the chamber; you can stop here for that reading < show them the "pause here for post-chamber vitals" sign on the floor>. We will then get a measurement after you finish doffing and lastly, we will get your vitals after you fill out the post-simulation questionnaire. (This is done to ensure that the participants' parameters are moving back towards baseline and are within a safe range before they leave the study site).

Now I'll show you around the chamber.

CHAMBER

This is the simulated **ETU** or Ebola treatment unit where all the tasks will be performed.

- The noise and temperature you hear now will be the same when you come in to do your tasks.

General Equipment Overview

- Here is your **working station table**: equipped with hand sanitizer and extra gloves.
- **Waste Bins:** the black waste bins are for plastic and paper waste; the yellow bin is for sharps and the red bin is for soiled waste. For example, any dressing or equipment that has been contaminated or has come in direct contact with the patients' blood or other bodily fluids. Be sure to carefully segregate any dispose of any of this hazardous material.
- There are 2 tables located closer to the patients' bed side.
- Feel free to move the tables around or turn on any of the lights if you want.
- There are 2 chamber windows and 2 cameras mounted on the walls that will record the tasks and help with our observation.
- The **yellow lines** on the floor indicate which patients are **suspected** of having Ebola and the **red line** indicates a **confirmed** Ebola case. So for patients in beds #1 and #2, you will need to take blood samples for laboratory testing.

Simulation Walkthrough

I'll give you a brief run through of how the simulation will go. <walk the participant through each step > When you first enter the chamber, you will be carrying your equipment tray. You can put your tray on the working station table. Make sure to bring whatever you need from the working station table to your patient's bedside tables.

After you have organized your equipment let us know when you are ready and we will read you the **case report** of your first patient. After we have finished reading you the scenario, you can begin the task.

<go to bed #1> For the patient in bed #1 you will need to insert a peripheral IV, provide fluids and take 2 blood samples. The veins on this patient might be hard to find. So, if you are having trouble you can use this the diagram to help guide you.

After you have completed the task or if you would like to move on, let us know and we will read you the case report for your next patient.

<go to bed #2> For the patient in bed #2, you will need to insert a mid-line catheter,

provide fluids and take 2 blood samples. Here is your surgical gown and drape.

<go to bed #3> Lastly, for the patient in bed #3, ensure the airway is maintained, suction the oral cavity, use airway adjuncts, administer appropriate intubation related medications and verify proper endotracheal tube placement. Again, this is a confirmed Ebola case, so you do not need to collect samples for this patient.

I'll show you some of the equipment for this procedure:
Bottom of bed: Here we have a bag-vale ventilator, a towel and a kit of Oro-Pharyngeal Airways (OPAs) of various sizes. **On wall:** Here is the ventilator, which you can turn on with this switch right here. Here is the ventilator connector, airway mask, and suction tube.

All this equipment isn't actually functional and are just **props** for the simulation.

Other Expectations

Even though this is a simulation, we want you to treat the patients as you normally would. Be sure to maintain normal **patient etiquette**, such as introducing yourself to the patient, explaining the procedure out loud and obtaining informed consent.

While in the red zone, it is critical to maintain proper **infection control** practice at all times. For example, please ensure you frequently perform proper **hand hygiene,** follow appropriate **sanitization procedures** and adhere to proper **sharps management** practice.

Speaker System

During the simulation, we will be outside the chamber and we will communicate with you via a **speaker** and microphone system that you will wear on top of your PPE. If for any reason, your microphone isn't working and you need our attention, just give us the thumbs down signal and we will come in to assist you.

Very important: While in the chamber, please try your best to think out loud and describe every little thing you are doing. This will be a big help to our observers and will ensure accurate data collection.

Blood Pressure and Thermal Scale Pause

Lastly, every **10 minutes**, we will ask you to stop what you are doing and take a brief pause so we can take your blood pressure. At this time, we will also ask you how you are feeling based on this thermal comfort scale (show the participants all locations of the thermal scale posters on the chamber walls). We will need you to give us a number from 1 to 13. 1 being 'you're so cold that you feel helpless', and 13 being 'you are so hot that you feel sick or nauseous'. The thermal scale rating is purely a rating of heat. It's not a measure of how uncomfortable you are in general; it is solely referring to how hot you feel.

Safety

For safety purposes, if you have a thermal rating of 12 or higher we typically suggest exiting the chamber. We will also be closely monitoring your vitals from outside the chamber. If any of these measurements surpass our any of our pre-determined safety cut-off points, we will ask you to stop whatever you are doing and then exit the chamber. Your safety and wellbeing are our top priority, so if at any point you feel unwell or uncomfortable or want to exit the chamber, please don't hesitate to let us know.

EQUIPMENT

Here is the physiological equipment that we will be using to measure your vitals during the simulation. To ensure your safety, we will be continuously monitoring this equipment and we will terminate the simulation if any of these measurements surpass a safe range.

1. Equivital
The Equivital is the first piece of equipment that will go on. It will record your heart rate and breathing rate. <Demonstrate > The garment goes over the left shoulder and clips at the front.

Sensors are along the bottom of garment. The sensor has to be in contact with the skin in order to work, so let us know if it's coming loose at any time (women) and make sure that no undergarments are blocking the sensor's contact with the skin.

Also when we put it on we have to wet the sensors first, so it might feel a little cold at first.

2. Thermistors
The thermistors will go on after the Equivital. This device will record your skin temperature.

< Show picture> There are 4 leads, attached to data logger. The data logger will be on a belt, worn around your waist. The leads will be attached along your right side on your shoulder, chest, thigh and side of your shin (about half way between knee and ankle).

Skin preps will be used to prepare the surface and the thermistors will be attached with medical tape. If you are allergic or sensitive to the adhesives be sure to let us know. (If the participant is hairy) To avoid discomfort when pulling the tape off and to secure the leads better, we recommend shaving a small patch of hair. Is that all right with you?

3. Thermobuttons
The Thermobuttons will measure temperature of the air inside the coveralls. They have already been taped to the inside of coverall at the same level of each thermistor.

4. Blood pressure Cuff
This will go on the inside of your coveralls. We are going to put the cuff on your non-dominant hand, so it doesn't interfere with your task performance. Are you right handed or left handed?

Every 10 min, we will ask you to pause for a blood pressure reading and the cuff will begin to inflate. It usually takes around 20- 30 seconds to get an accurate reading. The cuff doesn't work if you are moving around, so during this time, please try to remain as still as possible. Once we get a reading we will tell you when it is okay to proceed.

The blood pressure cuff communicates with a phone app via Bluetooth. The blue light indicates that the two devices are connected and a flashing blue light indicates that the devices are pairing. < demonstrate> If this blue light turns off while you are in the chamber, we will ask you to push this start/stop button until the blue light begins flashing.

It is often difficult to get a signal through the metal walls of the chamber and it can sometimes take up to 4 or 5 minutes for the devices to connect. So after you press the button and see the flashing blue light, you can continue treating your patients while the devices pair. We will notify you when we when the devices have reconnected and we will then ask you to pause what you are doing for a blood pressure reading.

5. Speaker system
This is the speaker headset we will use to communicate with you while you're in the chamber. After you finish donning, we will attach the system to the back of your apron and will perform a sound check before you enter the chamber.

<Demonstrate> If you want to adjust the volume while you're in the chamber. You can unclip it from the back of your apron and turn this dial to adjust the volume.

As we said before, if you're having trouble with your microphone, just give us a thumbs down and that will be our sign to come in and assist you.

6. Supplies Tray
Lastly, this tray contains all the supplies you will need for each task. <Show which tray for which task>

After you finish donning your PPE, you are expected to bring this tray and all the materials you need. You can't exit the red zone once you have entered, so make sure nothing is missing from the tray and inspect the equipment to make sure everything is functional.

Also this equipment might be a little different from the equipment you are used to so you might want to take the time to familiarize yourself with the supplies. You can do this now before we move on. Take your time.

Okay, lastly, I'll show you the changeroom.

CHANGEROOM

We ask that for safety reasons please do not lock the door.

You can leave any clothes and personal belongings in the drawers or hanging on the hooks. If you didn't bring your own, we have a clean pair of scrubs and socks for you to use.

These bins are for after the simulation. You can put your dirty scrubs into the dirty scrubs bin and then carefully remove the physiological equipment and place it in this bin after simulation. If you want some help taking off the equipment, just let us know and we would be happy to assist you.

As we mentioned in the participant information PowerPoint, we need a way to approximate the amount of water you lose during this simulation. To do that, we will need to take a weight with no clothing, before and after the simulation. This is the scale that we are going to use. To protect your privacy, you will be in this room alone with the doors closed during the weighing. We will be able to read the result from out here (show them the scale that is out of the room).

After you have removed all your clothing step up on the scale and stand with your feet wide apart <get up on scale and demonstrate>**.** Let us know when you're on the scale and try to stand as still as possible, it sometimes takes a couples of seconds to get a measure.

After we get a reading, you can put just your undergarments back on (bra and underwear). Let us know when you are ready and one of us will come in and attach the Equivital and thermistors.

After the equipment is attached, you can put on your scrubs and a pair of socks and come out of the changeroom when you're ready.

I know this is a lot of information to remember, so if you get lost we have step by step instructions up here on the wall for you to reference.

So that concludes our tour. Any questions before we move on to some paperwork?

ADMINISTRATION

You completed the PAR-Q and pre-simulation questionnaire online. Since you filled out the PAR-Q, has there been any recent changes to your health that we should know about?

Okay so here is the study consent form. Take as much time as you need to read it over and feel free to ask us any questions. When you're done reading it, please fill in and sign all the yellow highlighted sections.

Did you have a chance to watch the study videos? Would you like to watch any of them again before we begin the simulation?

PRE-SIMULATION VITALS

Come have a seat and we'll take a set of vitals

1. BP cuff: SBP, DBP, HR
2. Tympanic temperature - Does that feel like it's in properly?
If no → (readjust or allow them to guide you or allow the participant to do it themselves)
3. Chest – To make sure the Equivital vest fits you properly we need to get a chest measurement. Can you please stand up and raise your arms to shoulder height.

WEIGHT + EQUIPMENT

Now let's get your weight measurement.

Please remove all clothing articles and let us know when you are standing on the scale.

<record weight in kg, one decimal >

Ok. You can now put your undergarments garments back on. Call out to us when you're ready and **(study team member's name)** will come in and attach the equipment.

<Put on Equivital → wet the pads and check function on the app>

<Put on thermistors → skin prep, shave (if necessary) and tape >

Move around, is anything pulling or feel uncomfortable?

Ok. You can now put on your scrubs and socks

Take your time and come out whenever you are ready.

PRE-DONNING

Height: Before you begin donning we need to get your height. Please stand up straight with your back against the wall.

< attach BP cuff and get them to locate the start/stop button>

All right, you can now begin to don your PPE.

All right, you can now begin to don your PPE. <record the time in the CRF - HH:MM: SS>

<participant dons>

POST-DONNING

You can have a seat, we are going to check your vitals again before you enter the chamber.

1. Blood Pressure: Try to keep your arm still while the blood pressure cuff inflates.
2. Tympanic Temperature - We also need your temperature reading <lift coverall hood and insert thermometer> Does that feel like it's in properly?
If no → (readjust or allow them to guide you or allow the participant to do it themselves)

< plug in chamber ACRs>

<check Equivital app>

Speaker
Here is your speaker headset. Is that comfortable? Can you hear me? How's the volume?

Just a reminder, please try your best to describe everything you are doing or planning on doing out loud for our observers.

ID #
If the participant gave permission to be recorded in their consent form:
Lastly, we are going to write your participant number on the front of your apron and back of your coverall.
Are you ready to enter the chamber?

<record the time they enter the chamber with their equipment - HH:MM:SS>

EXITS CHAMBER

Please pause for a quick temperature and blood pressure reading
< measure vitals (BP, HR, Temp)>
.. okay you may proceed

POST-DOFFING

Take a seat and we are going to take another set of vitals.
< measure vitals (BP, HR, Temp)>

WEIGHT

We will need your post-simulation weight measurement.

Once you enter the change room please put your scrubs into the dirty scrubs bin and carefully remove all your physiological equipment and place them into the equipment bin. Once everything is removed, let us know when you are standing on the scale

Would you like to use the shower facilities after the study?

<record weight in kg, one decimal >

You can now change back into your clothes. Meet us outside when you are ready.

If they wanted to shower: or if you prefer there is a towel in the bottom drawer and flip flops by the wall. When you're done one of us can show you to the facilities.

< participant exits change room> Please come take a seat over here. Would you like any water or snacks before we measure your vitals? < measure vitals (BP, HR, Temp)>

< give the participant the post-simulation questionnaire>

< measure vitals (BP, HR, Temp)>

RECEIPT
Lastly, to thank you for your time we give our participants $67 and reimburse them for any parking or travel related expenses. Did you have any other travel expenses that we can reimburse you for?

Can we get your name and signature at the bottom of this receipt.

I think that is all we need for you today. Thanks again for taking the out of your busy schedule to participate in this study. Take as much time as you need to rest and rehydrate. We will escort you out when you are ready.

File S4: Patient vignettes

Participant enters chamber

Leave your equipment tray on the working station. Bring any equipment you need with you to the patient's table by bed #1. Let us know when you are ready and we will read you your first patient's case report.

TASK 1- PERIPHERAL IV INSERTION

Michael is a 30 year old male. He was admitted yesterday after being evacuated from the Ebola zone in Sierra Leone. He reported a 2-day history of fever, headache, joint pain and diarrhea. This morning he vomited twice and had three episodes of diarrhea. Michael complains of abdominal pain and hiccups. He is alert and can obey commands.

After a vitals assessment you find that:

- HR: His heart rate is 124 beats per minute.
- BP: His blood pressure is 90 over 40.
- Temp: His temperature is 39 degrees Celsius.
- RR: and his respiratory rate is 18 breaths per minute.

Michael has not yet received any treatment and no laboratory tests have been done.

Do you want me to read this scenario again or do you need me to repeat any information?

Please remember to try your best to describe what you are doing out loud. You can now begin treating the patient whenever you are ready.

If the participant asks any questions that are not mentioned above → say that is unknown.

Every 10 min → Take Blood Pressure and inquire about Thermal Scale

Please stop what you are doing and pause for a blood pressure reading. Press the START/STOP button on the blood pressure cuff to start the reading. While it is inflating, try your best to remain still. When it is finished, you can read out the results or you can move closer to the observation window and show us the screen * record HR and BP

Great thanks, and from 1-13, where are you on the thermal comfort scale.

If Thermal Scale ≥ 12

→ Based on our safety protocol we recommend leaving the chamber if you are at a thermal scale level of 12 or higher, would you like to exit now?

* Note it as a health-concerning incident (H) in the CRF
* If they wanted to continue, remember to ask why after they exit and note the reason in the CRF.

TASK 2- MID-LINE CATHETHER INSERTION

This is Lucy, a 35-year-old female who was admitted 2 days ago with vomiting, fever, headache, and generalized weakness. Upon admittance, she had mild diarrhea with about 2 episodes per day. During the first two days, she was able to eat and drink and was managed with Oral Rehydration Salts.

This morning you notice Lucy has become increasingly confused and is now unable to leave his bed. She informs you that she has passed little urine in the last 24 hours and her diarrhea has increased to more than 5 episodes a day. She is vomiting and has completely lost her appetite.

After a vitals assessment you find that:

- HR: Her heart rate is 110 to 120 beats per minute.
- BP: Her blood pressure was not done but his extremities are cold and clammy.
- Temp: Her temperature is 39 degrees Celsius.
- RR: Her respiratory rate is 12 breaths per minute.
- O2: and her oxygen saturation is 96%.

Lucy has been treated with Oral Rehydration Salts.
No laboratory tests have been done.

Do you want me to read the scenario again or do you want me to repeat any information?

Please remember to try your best to describe what you are doing out loud. You can now begin treating the patient whenever you are ready.

If the participant asks any questions that are not mentioned above → that is unknown

---

Every 10 min → Blood Pressure and Thermal Scale Pause

Please pause for a blood pressure reading. Press the start/stop button and try your best to remain still while the cuff is inflating. * record HR and BP

Great thanks, and from 1-13, where are you on the thermal comfort scale.

If Thermal Scale ≥ 12

→ Based on our safety protocol we recommend leaving the chamber if you are at a thermal scale level of 12 or higher, would you like to exit now?

* Note it as a health-concerning incident (H) in the CRF
* If they wanted to continue, remember to ask why after they exit and note the reason in the CRF.

TASK 3 – INTUBATION

This man was dropped off by an ambulance a few hours ago. He was not accompanied by relatives and the only background information that the ambulance attendants provided is that his name is Steven, he is in his late 20s and he lost his wife to Ebola one week ago. He was transferred to this ward after his laboratory tests came back positive for Ebola Virus Disease.

He is unable to speak and is soaked in a pool of diarrhea and vomitus. After a vitals assessment you find that:

- HR: His heart rate is 90 beats per minute.
- BP: His blood pressure was not done but his extremities are cold and clammy.
- Temp: His temperature is 38 degrees Celsius.
- Breathing: The patient is in respiratory distress and his airway appears blocked.
- Consciousness: The patient is semi-conscious and is a 10 on the Glasgow Coma Scale.
- RR: His respiratory rate is 30 breaths per minute.
- O2: and his oxygen saturation is 82%.

This patient has not yet received any treatment and it is not certain what medications he received before arrival.

Do you want me to read this scenario again or do you need me to repeat any information?

Please remember to try your best to describe what you are doing out loud. You can now begin treating the patient whenever you are ready.

If the participant asks any questions that are not mentioned above → that is unknown

---

Every 10 min → Blood Pressure and Thermal Scale Pause

Please pause for a blood pressure reading. Press the start/stop button and try your best to remain still while the cuff is inflating. * record HR and BP

Great thanks, and from 1-13, where are you on the thermal comfort scale.

If Thermal Scale ≥ 12

→ Based on our safety protocol we recommend leaving the chamber if you are at a thermal scale level of 12 or higher, would you like to exit now?

* Note it as a health-concerning incident (H) in the CRF
* If they wanted to continue, remember to ask why after they exit and note the reason in the CRF.

File S5: Post- Simulation Questionnaire

Health Questions

1. During the simulation did you experience any of the following? **(Check all that apply)**

☐ Nausea
☐ Heart racing
☐ Shortness of breath
☐ Muscle trembling, twitches or cramps
☐ Dizzines or lightheadedness
☐ Feeling uncomfortably hot
☐ Exhaustion
☐ Difficulty focusing or thinking clearly
☐ Frustration
☐ Anxiety
☐ Hunger
☐ Thirst
☐ None
☐ Other: ____

PPE Questions

2. To what extent did each protective item interfere with your ability to **provide effective patient care** (i.e. interfering with your ability to perform tasks requiring manual dexterity such as finding a vein)

	**No Reduction** in ability to provide care	**Minor Reduction** in ability to provide care	**Manageable Reduction** in ability to provide care	**Major Reduction** in ability to provide care	**Unable** to provide care
Face Shield	☐	☐	☐	☐	☐
Face Mask	☐	☐	☐	☐	☐
Coverall Hood	☐	☐	☐	☐	☐
Coverall	☐	☐	☐	☐	☐
Non-Sterile Gloves	☐	☐	☐	☐	☐
Sterile Gloves	☐	☐	☐	☐	☐
Sterile Gown	☐	☐	☐	☐	☐

Additional comments:

3. To what extent did each PPE item affect your personal well-being with regards to **HEAT?**

	Not Hot at all	Warm but comfortable	Tolerably Hot	Uncomfortably Hot	Unbearably Hot
Face Shield	☐	☐	☐	☐	☐
Face Mask	☐	☐	☐	☐	☐
Coverall Hood	☐	☐	☐	☐	☐
Coverall	☐	☐	☐	☐	☐
Non-Sterile Gloves	☐	☐	☐	☐	☐
Sterile Gloves	☐	☐	☐	☐	☐
Sterile Gown	☐	☐	☐	☐	☐
Boots	☐	☐	☐	☐	☐

Additional comments:

4. Excluding considerations of heat, how **COMFORTABLE** did you find each of the protective items to wear?

	Very Comfortable	Somewhat Comfortable	Neither Comfortable nor Uncomfortable	Somewhat Uncomfortable	Very Uncomfortable
Face Shield	☐	☐	☐	☐	☐
Face Mask	☐	☐	☐	☐	☐
Coverall Hood	☐	☐	☐	☐	☐
Coverall	☐	☐	☐	☐	☐
Gloves	☐	☐	☐	☐	☐
Non-Sterile Gloves	☐	☐	☐	☐	☐
Sterile Gown	☐	☐	☐	☐	☐
Boots	☐	☐	☐	☐	☐

Additional comments:

5. At any point during the simulation did you feel that the PPE was significantly reducing your performance? (Check all that apply)

☐ PPE did not interfere with my performance
☐ As soon as I walked into the simulation area
☐ During IV task
☐ During mid-line task
☐ During intubation task
☐ Not Sure
Other: ____

**Additional comments:**

6. What element(s) of the study did you feel <u>least</u> confident about (i.e. donning/ doffing, equipment, tasks)?

7. Did you experience any difficulties or accidents while putting on, wearing, or removing your PPE?

8. During the simulation did any of your PPE rip, tear, or break? (i.e. coverall, gloves, apron)<br>If yes: What item of PPE? When during the simulation did this occur? Where was the rip located?

9. Did you experience any accidental needle pricks or other sharps injuries?

10. At any point in the simulation did you experience any patient fluid splashes onto your PPE?<br>(i.e. mannequin blood splashed onto face shield)

11. In your opinion, what improvements could be made to the PPE?

12. Do you have any additional comments or considerations related to the PPE you were wearing that were not asked above?

Task Questions

13. At any point during the simulation did you feel like you wanted to leave the chamber OR feel like you needed to take a break in between tasks?<br>If **yes**, please describe why. At what point during the simulation did this occur?<br>*** this question will be deleted post- pilot**

14. Compared to what you expected, how challenging did you find each of the study tasks?

	**Much Easier** than I expected	**Slightly Easier** than I expected	**The Same** as I expected	**Slightly More Difficult** than I expected	**Much More Difficult** than I expected
Donning PPE	☐	☐	☐	☐	☐
Doffing PPE	☐	☐	☐	☐	☐
IV Insertion Task	☐	☐	☐	☐	☐
Mid-line Catheter Task	☐	☐	☐	☐	☐
Intubation Task	☐	☐	☐	☐	☐
Triple Packaging Samples	☐	☐	☐	☐	☐

**Additional Comments:**

15. How much effort did you put into each task? (Circle **one** number for each task)

1 = No effort   5 = Maximum effort (i.e. I tried as hard as I could)

Donning	1	2	3	4	5
Doffing	1	2	3	4	5
IV Insertion Task	1	2	3	4	5
Mid-line Catheter Task	1	2	3	4	5
Intubation Task	1	2	3	4	5

Additional Comments:

16. How well do you think you performed on each task? (Circle **one** number for each task)
1 = I think I performed very poorly **5 = I think I performed perfectly**

Donning	1	2	3	4	5
Doffing	1	2	3	4	5
IV Insertion Task	1	2	3	4	5
Mid-line Catheter Task	1	2	3	4	5
Intubation Task	1	2	3	4	5

Additional Comments:

17. How nervous or anxious did you feel during each task? (Circle **one** number for each task)
1 = Not at all **5 = Extremely**

Donning	1	2	3	4	5
Doffing	1	2	3	4	5
IV Insertion Task	1	2	3	4	5
Mid-line Catheter Task	1	2	3	4	5
Intubation Task	1	2	3	4	5

Additional Comments:

Video Questions

18. How many times did you watch each of the study videos?

	0	1	2	3	4 or more
Donning Video	☐	☐	☐	☐	☐
Doffing Video	☐	☐	☐	☐	☐
IV Insertion Video	☐	☐	☐	☐	☐
Mid-line Catheter Video	☐	☐	☐	☐	☐
Intubation Video	☐	☐	☐	☐	☐
Triple Packaging Video	☐	☐	☐	☐	☐

19. When did you last watched each study video?

	Today (at the study site)	Today (before arriving at the study site)	Yesterday	More than 3 days ago	More than a week ago	I never watched it
Donning Video	☐	☐	☐	☐	☐	☐
Doffing Video	☐	☐	☐	☐	☐	☐
IV Insertion Video	☐	☐	☐	☐	☐	☐
Mid-line Catheter Video	☐	☐	☐	☐	☐	☐
Intubation Video	☐	☐	☐	☐	☐	☐
Triple Packaging Video	☐	☐	☐	☐	☐	☐

General Questions

20. In your opinion, what improvements could be made regarding the whole simulation process?

21. After this simulation, do you think your interest to help in future outbreaks has increased or decreased? Please explain why:

22. How did you find the length of this questionnaire?
☐ Too short
☐ Somewhat short
☐ Just right – not too short or too long
☐ Somewhat too long
☐ Too long

23. Any additional or final comments?

File S6-1 Case Report Form

CRF- EBOLA TREATMENT UNIT SIMULATION STUDY					
1	Study Date:____________/____________/____________(DD/MM/YYYY)				
2	Participant's Age	______________ (years)			
3	**I_I__I:I__I__I**	Green Zone Temperature________.____(°C)			Green Zone Humidity_____________(%)
4	Did the participant watch any of the videos?<br>(Note how many times they watched each one)<br>Start: **I__I__I:I__I__I** End: **I_I_I:I_I_I**		Y	N	_____Donning_____Doffing_____Triple Packaging<br>____IV______Mid-line Catheter______Intubation

VITALS #1: PRE-SIMULATION				
5	**I_I__I:I__I__I**	**SBP** (mmHg):____________**DBP** (mmHg):_____________**HR** (beats/min): ______________		
6	**Tympanic Temperature (°C)**		**1st Reading:**________.____	**2nd Reading:**________.____
7	Chest Size (cm)	____________	Equivital Size	____________
☐ **Wet Equivital Sensors**				

CHANGEROOM				
8	**I_I__I:I__I__I**	Weight (kg)<br>(dry, unclothed weight)	**1st Reading:**________.____	**2nd Reading:**__________.____
9	Height (cm)	____________		
☐ **Attach BP cuff** before donning (non-dominant arm)			☐ Ensure the Equivital chest unit is working before donning	

Note: Letter, Time and Description
R = Reminded **I** = Interruption **P** = BP, HR and Thermal Scale Pause
B = Breach in PPE **N** = Near-miss incident **H** = Health incident

Participant ID: ____________ Observer's Initials: ____________ CRF Version 19, *March 12th, 2019 (Pilot Version)*

DONNING

10	**Time assessor says begin donning**	**I__I__I:I__I__I:I__I__I**

#	Done Well	Done	Not Done	Not Sure	Steps	R	I	P	B	N	H	Times and Descriptions
11	2	1	0	?	**Inspects PPE**<br>2 = 3 or more PPE items							
12		1	0	?	**Sanitizes Hands**<br>**– before putting on any PPE items**							
13		1	0	?	**Rubber Boots**							
14		1	0	?	**Gloves**							
15		1	0	?	**Coverall**							
16		1	0	?	**Cuffs:** slips both thumbs							
17	2	1	0	?	**Face Mask**<br>1. Places one strap above the ears<br>2. Places other strap below the ears<br>3. Performs a seal check							
18	2	1	0	?	**Face Shield:** inside hood							
19	2	1	0	?	**Coverall Hood**							
20		1	0	?	**Apron**							
21	2	1	0	?	**Second Gloves**<br>over the coverall cuff							
22	2	1	0	?	**Mirror Check**<br>OR asks a buddy to help							

Record the time when the participant finishes donning **I__I__I:I__I__I:I__I__I**

R: ________ **I:** ________ **B:** ________ **N:** ________ **H:** ________

Note: Letter, Time and Description

R = Reminded **I** = Interruption **P** = BP, HR and Thermal Scale Pause
B = Breach in PPE **N** = Near-miss incident **H** = Health incident

VITALS #2: POST-DONNING			
23	I_I__I:I__I__I	**SBP** (mmHg):___________ **DBP** (mmHg):_____________ **HR** (beats/min): ____________	
24	**Tympanic Temperature (°C)**	**1st Reading:_________._____**	**2nd Reading:_________._____**

POST- DONNING CHECKLIST

- ☐ **Speaker Headset**
- ☐ **Participant ID:** write on the front of apron and back of the coverall
- ☐ **Turn off donning camera** ☐ **Plug** in the ACRs to get the temperature and humidity readings of the chamber
- ☐ **Ready?** Ask participant if they are ready to enter the chamber
- ☐ **Chamber temperature________.____**(°C), Chamber Humidity____________(%). Time: **I_I__I:I__I__I**

TASK 1: PIV INSERTION				
25	**Participant enters ETU with equipment tray**	☐ Done	☐ Not Done	**I_I__I:I__I__I:I__I__I**
26	**Time the assessor starts reading the scenario**			**I_I__I:I__I__I:I__I__I**
27	**Did the participant ask the assessor to repeat anything**	Y	N	
28	**Time the assessor finishes reading the scenario**			**I_I__I:I__I__I:I__I__I**

#	Done well	Done	Not Done	Not Sure	Steps	R	I	P	B	N	H	Times and Descriptions
29		1	0	?	**Introduces Self**<br>to the patient							
30		1	0	?	**Sharps Bin:**<br>places within arm's reach							N- I_I__I:I__I__I
31		1	0	?	**Sanitizes Hands**<br>**- before touching the patient**							
32		1	0	?	**Tourniquet**							**I_I__I:I__I__I:I__I__I**
33		1	0	?	**Feels for Vein**							
34	2	1	0	?	**Sanitizes Procedural Site**<br>**- before inserting the needle**							

Note: Letter, Time and Description
R = Reminded **I** = Interruption **P** = BP, HR and Thermal Scale Pause
B = Breach in PPE **N** = Near-miss incident **H** = Health incident

						R	I	P	B	N	H	
35	2	1	0	?	**Inserts Needle into Vein**							I_I__I:I__I__I:I__I__I
36		1	0	?	**Confirms needle is in vein**<br>1. Backflow of blood is visible in the catheter<br>2. Aspirates blood with syringe<br>3. Flushes line with sterile water							I_I__I:I__I__I:I__I__I
37	How many **needle pricks?** (Tally):_________________________ **Total #:**_________				How many **cannulas?** (Tally):_______________ **Total #:** _________							
38	2	1	0	?	**Removes Tourniquet**<br>**- immediately after vein access**							I_I__I:I__I__I:I__I__I
39		1	0	?	**Disposes of Needle**<br>immediately in sharps bin							
40		1	0	?	**Secures Cannula:**<br>**with Tape or Tegaderm**							
41	2	1	0	?	**Obtains Blood Samples**							I_I__I:I__I__I
42	2	1	0	?	**IV Fluid Therapy**<br>2 = connects AND checks the line							I_I__I:I__I__I
43		1	0	?	**Applies end cap OR**<br>**clips cannula**							
44	2	1	0	?	**Disinfects Blood Tubes**							
45		1	0	?	**Wraps Blood Tubes with Napkin**							
46		1	0	?	**Sanitizes Hands- after disinfecting AND before placing blood tubes in secondary tubes**							
47	2	1	0	?	**Secondary Tubes** (primary tubes inside)							
48	2	1	0	?	**Disinfects any Blood from Site**							N/A

Note: Letter, Time and Description
R = Reminded **I** = Interruption **P** = BP, HR and Thermal Scale Pause
B = Breach in PPE **N** = Near-miss incident **H** = Health incident

						R	I	P	B	N	H	
49	2	1	0	?	**Waste:** Properly segregates and disposes of all waste							
50		1	0	?	**Sanitizes <u>Outer</u> Gloves - before putting on a new pair of gloves**							
51	2	1	0	?	**New Gloves:** Changes outer gloves							
Time when participant finished task						I_I__I:I__I__I						

R:_________ I:_________ P:_________ B:_________ N:_________H: _________

TASK 2: MID-LINE CATHETER INSERTION												
52	**Time the assessor starts reading the scenario**											**I_I__I:I__I__I:I__I__I**
53	**Did the participant ask the assessor to repeat anything?**					Y			N			
54	**Time the assessor finishes reading the scenario**											**I_I__I:I__I__I:I__I__I**
#	**Done well**	**Done**	**Not Done**	**Not Sure**	**Steps**	R	I	P	B	N	H	Times and Descriptions
55		1	0	?	**Introduces Self**							
56		1	0	?	**Sharps Bin:** places within arms' reach							N/A
57		1	0	?	**Sanitizes Hands - before touching the patient**							
58	2	1	0	?	**Sanitizes Procedural Site - before putting on the drape**							
59	2	1	0	?	**Sterile Field** opens items in a sterile manner (without touching them)							**Start: I_I_I:I_I_I**<br>**End: I_I_I:I_I_I**

<u>Note: Letter, Time and Description</u>
R = Reminded **I** = Interruption **P** = BP, HR and Thermal Scale Pause
B = Breach in PPE **N** = Near-miss incident **H** = Health incident

												_Gown _Drape _Syringe<br>__Gauze _ Sterile Gloves<br>__Mid-Line Kit ___Tegaderm
						R	I	P	B	N	H	
60		1	0	?	**Sanitizes Hands**<br>**- before removing their gloves**							
61	2	1	0	?	**Removes Outer Gloves**							
62	2	1	0	?	**Gown**							
63	2	1	0	?	**Sterile Gloves**<br>**– cuffs over gown**							
64	2	1	0	?	**Drape**							**Start: I__I__I:I__I__I**<br>**End: I__I__I:I__I__I**
65		1	0	?	**Sanitizes Procedural Site**<br>**- after putting on the drape and before giving Lidocaine**							
66	2	1	0	?	**Flushes the Catheter Lumens**							
67	2	1	0	?	**Lidocaine**							
68		1	0	?	**Disposes of Needle**<br>immediately in sharps bin							N- I_I_I:I_I_I
69	2	1	0	?	**Inserts Needle into Vein**							**I_I_I:I_I_I:I_I_I**
70		1	0	?	**Confirms needle is in vein by doing any one of the following:**<br>1. Aspirates blood with syringe<br>2. Line is flushed with sterile water.<br>3. Backflow of blood is visible in the catheter.							**I_I_I:I_I_I:I_I_I**
71	How many **needle pricks?** (Tally):_________________________ **Total #:** _______											
72		1	0	?	**Guide Wire**							

Note: Letter, Time and Description

R = Reminded **I** = Interruption **P** = BP, HR and Thermal Scale Pause
B = Breach in PPE **N** = Near-miss incident **H** = Health incident

73		1	0	?	**Removes Needle**							
74		1	0	?	**Disposes of Needle** <u>immediately</u> in sharps bin							N- I_I__I:I__I__I
75		1	0	?	**Introducer and Dilator**							
						R	I	P	B	N	H	
76	2	1	0	?	**Removes Guide Wire**							
77		1	0	?	**Removes Dilator**							
78	2	1	0	?	**Catheter**							**I_I__I:I__I__I**
79	2	1	0	?	**Removes Peel-away Introducer**							
80		1	0	?	**Removes Obturator**							
81	2	1	0	?	**Aspirates Blood** from each lumen and flushes them							
82		1	0	?	**Secures Catheter**							
83	2	1	0	?	**Obtains Blood Samples**							**I_I__I:I__I__I**
84	2	1	0	?	**4-Way Stopcock**							
85	2	1	0	?	**IV Fluid Therapy** 2 = connects AND checks the line							**I_I__I:I__I__I**
86		1	0	?	**End Cap**							
87	2	1	0	?	**Disinfects Blood Tubes**							
88		1	0	?	**Wraps Blood Tubes with Napkin**							
89		1	0	?	**Sanitizes Hands- <u>after</u> disinfecting AND <u>before</u> placing blood tubes in secondary tubes**							

<u>Note: Letter, Time and Description</u>
R = Reminded **I** = Interruption **P** = BP, HR and Thermal Scale Pause
B = Breach in PPE **N** = Near-miss incident **H** = Health incident

90	2	1	0	?	**Secondary Tubes**							
91	2	1	0	?	**Disinfects Secondary Tubes**							
92	2	1	0	?	**Disinfects any Blood from Site**							N/A
93		1	0	?	Applies transparent film dressing							
						R	I	P	B	N	H	
94	2	1	0	?	**Removes Drape**							**I_I__I:I__I__I**
95		1	0	?	**Sanitizes Hands**<br>**- after removing the drape**							
96	2	1	0	?	**Removes Sterile Gloves**							
97		1	0	?	**Sanitizes Hands**<br>**- after removing sterile gloves**							
98	2	1	0	?	**Removes Gown**							
99	2	1	0	?	**Waste:** Properly segregates and disposes of all waste							
100		1	0	?	**Sanitizes Hands**<br>**- after waste segregation OR**<br>**- before putting on new gloves**							
101	2	1	0	?	**New Gloves**<br>Changes outer gloves							
Time when participant finished task						**I_I__I:I__I__I**						

R:_________ I:_________ P:_________ B:_________ N:_________H: _________

**TASK 3: INTUBATION AND MECHANICAL VENTILATION**	
102	**Time the assessor starts reading the scenario** **I_I__I:I__I__I:I__I__I**

Note: Letter, Time and Description
R = Reminded **I** = Interruption **P** = BP, HR and Thermal Scale Pause
B = Breach in PPE **N** = Near-miss incident **H** = Health incident

103	**Did the participant ask the assessor to repeat anything?**					Y			N			
104	**Time the assessor finishes reading the scenario**											I_I__I:I__I__I:I__I__I
	**Done well**	**Done**	**Not Done**	**Not Sure**	**Steps**	R	I	P	B	N	H	Time and Description
105		1	0	?	**Introduces Self**							
106		1	0	?	**Sanitizes Hands<br>- before touching the patient**							
						R	I	P	B	N	H	
107		1	0	?	**Removes the blockage<br>- before the airway mask**							
108		1	0	?	**Suctions the oral cavity<br>- before the airway mask**							
109	2	1	0	?	**Airway Mask**							I_I__I:I__I__I:I__I__I
110	2	1	0	?	**ETT Prep**<br>- Checks for air-leaks by inflating AND deflating the cuff							
111	2	1	0	?	**Stylet**<br>Inserts when the cuff is deflated							
112		1	0	?	**Checks Laryngoscope Light**							
113	2	1	0	?	**IV Fluid Therapy**<br>2 = connects AND checks the line							I_I__I:I__I__I
114	2	1	0	?	**Medications**							I_I__I:I__I__I
115		1	0	?	**Removes Airway Mask**							I_I__I:I__I__I:I__I__I
116	2	1	0	?	**OPA**<br>Correct size (yellow or green)							
117		1	0	?	**Ventilates with Bag-Valve**							

Note: Letter, Time and Description
R = Reminded **I** = Interruption **P** = BP, HR and Thermal Scale Pause
B = Breach in PPE **N** = Near-miss incident **H** = Health incident

118	2	1	0	?	**Laryngoscope**							
119	2	1	0	?	**Inserts ETT**							**First Attempt:** **I_I__I:I__I__I:I__I__I**
120		1	0	?	**Inflates Cuff**							
121		1	0	?	**Capnometer**							
122	2	1	0	?	**Confirms ETT Insertion**<br>1. Ventilates with bag-valve<br>2. Symmetrical chest expansion							**I_I__I:I__I__I:I__I__I**
123. How many **ETT insertion** attempts? (Tally):_______________						**Total #:** _______						
						R	I	P	B	N	H	
124	2	1	0	?	**Tapes ETT**							
125		1	0	?	**Connects Ventilator**							
126		1	0	?	**Turns Ventilator On**							
127	2	1	0	?	**Waste:** Properly segregates and disposes of all waste							
128		1	0	?	**Sanitizes Hands - before exiting the chamber**							
129	2	1	0	?	**New Gloves:** Changes outer gloves							
Time when participant finished task						**I_I__I:I__I__I**						

R:_________ I:_________ P:_________ B:_________ N:_________H: _________

**DOFFING**												
130	**Time the participant exits ETU**					**I_I__I:I__I__I:I__I__I**						
	**Done well**	**Done**	**Not Done**	**Not Sure**	**Steps**	R	I	P	B	N	H	Time and Description

Note: Letter, Time and Description
R = Reminded **I** = Interruption **P** = BP, HR and Thermal Scale Pause
B = Breach in PPE **N** = Near-miss incident **H** = Health incident

131		1	0	?	**Disinfects Boots**							
132		1	0	?	**Places Samples into Sealable Bin**							
133		1	0	?	**Sanitizes Hands – before Apron**							
134	2	1	0	?	**Apron**							
135		1	0	?	**Sanitizes Hands – after removing Apron**							
136	2	1	0	?	**Unzips Coverall**							
137	2	1	0	?	**Hood**							
138	2	1	0	?	**Coverall (Shoulders)**							
139	2	1	0	?	**Outer Gloves**<br>2 = Outer surface of gloves doesn't touch inside coveralls							
140	2	1	0	?	**Coverall (Remaining Bottom)**<br>2 = Inner gloves touch the inside of coveralls							
141	2	1	0	?	**Disposes of Coverall**							
142		1	0	?	**Sanitizes Hands – after Coverall**							
143	2	1	0	?	**Face Shield**<br>2 = Participant bends head slightly forward, pulls strap from back of head and closes eyes							
144		1	0	?	**Sanitizes Hands – after Face Shield**							
145	2	1	0	?	**Face Mask**							
146		1	0	?	**Sanitizes Hands- after Face Mask**							
147		1	0	?	**Hair Cover**							N/A

Note: Letter, Time and Description
R = Reminded **I** = Interruption **P** = BP, HR and Thermal Scale Pause
B = Breach in PPE **N** = Near-miss incident **H** = Health incident

148		1	0	?	**Sanitizes Hands – after Hair Cover**						N/A	
149	2	1	0	?	**Removes Inner Gloves**							
150	2	1	0	?	**Disinfects Boots**							
151	2	1	0	?	**Washes Hands**							
**Record the time when the participant finishes doffing**						**I_I__I:I__I__I:I__I__I**						
**R:_________ I:_________B:_________ N:_________H: _________**												

**VITALS #3: POST-DOFFING**			
152	**I_I__I:I__I__I**	**SBP** (mmHg):__________**DBP** (mmHg):___________**HR** (beats/min): _________	
153	**Tympanic Temperature (°C)**	**1st Reading:_________.___**	**2nd Reading:_________.___**

**CHANGEROOM**				
154	**I_I__I:I__I__I**	Weight (kg)	**1st Reading:** _________.___	**2nd Reading:_________.___**

**VITALS #4: POST-SIMULATION**			
155	**I_I__I:I__I__I**	**SBP** (mmHg):__________**DBP** (mmHg):___________**HR** (beats/min): _________	
156	**Tympanic Temperature (°C)**	**1st Reading:_________.___**	**2nd Reading:_________.___**

**GREEN ZONE #2**			
157	**I_I__I:I__I__I**	Green Zone Temperature (°C)________.___	Green Zone Humidity (%) ___________

**PARTICIPANT SAFETY**
**PERSONAL PROTECTIVE EQUIPMENT (PPE)**

Note: Letter, Time and Description
R = Reminded **I** = Interruption **P** = BP, HR and Thermal Scale Pause
B = Breach in PPE **N** = Near-miss incident **H** = Health incident

158	Y	N	Was there a **minor** PPE breach?	
	If **no**, go to question **160**			
	If **yes**, go to question **159**			
159			**Type of Minor Breach**	**Breach Description**
a)	Y	N	Malposition of face shield or face mask	Times:<br>**Total Number:** ________
b)	Y	N	Tear in coverall suit	Times:<br>Where:<br>**Total Number:** ________
c)	Y	N	Tear in gloves	Times:<br>Where:<br>**Total Number:** ________
d)	Y	N	Tear in apron	Times:<br>Where:<br>**Total Number:** ________
e)	Y	N	Attempts to adjust face shield or face mask	Times:<br>**Total Number:** ________
f)	Y	N	Touches/scratches any area around the head	Times:<br>**Total Number:** ________
g)	Y	N	Splash of blood onto PPE	Times:<br>**Total Number:** ________
h)	Y	N	Other: ________________________	Times:<br>**Total Number:** ________
**Total Number of Minor Breaches:** ____________				
160	Y	N	Was there a **major** PPE breach?	
	If **no**, go to question **162**			

Note: Letter, Time and Description
R = Reminded **I** = Interruption **P** = BP, HR and Thermal Scale Pause
B = Breach in PPE **N** = Near-miss incident **H** = Health incident

	If **yes**, go to question **161**			
161			**Type of Major Breach**	**Breach Description**
a)	Y	N	Needle stick or other sharps injury	Times:<br>**Total Number: ________**
b)	Y	N	Exposure of the skin, mouth, eyes or nose to patient's blood	Times:<br>**Total Number: ________**
c)	Y	N	Contact of the dirty gloves with the inside of the PPE , skin or mucous membranes	Times:<br>**Total Number: ________**
d)	Y	N	Other: ___________________________	Times:<br>**Total Number: ________**
**Total Number of Major Breaches: ____________**				

**NEAR-MISS INCIDENTS**				
162	Y	N	Was there a near-miss incident?	
	If **no**, go to question **164**			
	If **yes**, go to question **163**			
163			**Type of Near-Miss Incident**	**Near-Miss Incident Description**
a)	Y	N	Needle not placed immediately in sharps bin after completion of task	Times:<br>**Total Number: ________**
b)	Y	N	Recapping of needle	Times:<br>**Total Number: ________**

Note: Letter, Time and Description
R = Reminded **I** = Interruption **P** = BP, HR and Thermal Scale Pause
B = Breach in PPE **N** = Near-miss incident **H** = Health incident

c)	Y	N	Walking with an exposed needle or contaminated object (i.e. syringe containing blood, soiled drape, soiled gauze, soiled alcohol wipes)		Times:<br>**Total Number:** ________
d)	Y	N	Other: ________________________		Times:<br>**Total Number:** ________
**Total Number of Near-Miss Incidents:** ____________					
**HEALTH-CONCERNING INCIDENTS**					
164	Y	N	Was there a health-concerning incident?		
	If **no**, go to question **169**				
	If **yes**, go to question **165**				
165	Y	N	I__I__I:I__I__I	Did the participant have to exit the chamber due to this health-concerning incident? If the participant declines to exit the chamber, state the reason.	
166	Y	N	N/A	Did the participant **self-recognize** this health-concerning incident?	
167	Y	N	Did the participant surpass any of the physiological cut-off points?		
	If **no**, go to question **169**				
	If **yes**, go to question **168**				
168	What physiological cut-off did the participant surpass? (Check all that apply)				

			Time	Type of Physiological Cut-Off	Value
a)	Y	N	I__I__I:I__I__I	HR ≥ **85%** of HR max	
b)	Y	N	I__I__I:I__I__I	**30 ≤** HR **≥ 200 beats/minutes** (N.B: for the upper limit of HR, use individualized HRmax to prompt symptom screen**)**	
c)	Y	N	I__I__I:I__I__I	SBP ≤ **90 mmHg** (x 2 readings taken in succession)	
d)	Y	N	I__I__I:I__I__I	SBP drops **> 40 mmHg** below baseline	

Note: Letter, Time and Description
R = Reminded **I** = Interruption **P** = BP, HR and Thermal Scale Pause
B = Breach in PPE **N** = Near-miss incident **H** = Health incident

e)	Y	N	I__I__I:I__I__I	SBP **> 180 mmHg**	
169	Y	N	Was there any other type of incident **OR** was participant removed from the chamber for any other reason?		
	If **no**, then **form is complete**				
	If **yes**, go to question **170**				
170			**Other Types of Incidents**		
a)	Y	N	I__I__I:I__I__I	Assessor's discretion (e.g. participant couldn't focus and conduct tasks appropriately, incoherence with instruction, etc.)<br><br>**Comment:**	
b)	Y	N	I__I__I:I__I__I	The participant did not want to continue participating the study (at any point during the study).<br><br>**Comment:**	
c)	Y	N	I__I__I:I__I__I	Thermal scale ≥ **12** and the participant exited the chamber	
d)	Y	N	I__I__I:I__I__I	Thermal scale ≥ **12** and the participant did not exit the chamber<br>**Describe Why:**	
e)	Y	N	I__I__I:I__I__I	**Other:**	

Additional Notes:

<u>Note: Letter, Time and Description</u>
R = Reminded **I** = Interruption **P** = BP, HR and Thermal Scale Pause
B = Breach in PPE **N** = Near-miss incident **H** = Health incident

File S6-2: Instructions on how to fill the CRF

This instructor Guide (IG) explains how to fill in the CRF, it flows in the same particular order in which the CRF is laid out.

Acronyms:

BMI	Body Mass Index
CRF	Case Reporting Form
CO_2	Carbon dioxide
DBP	Diastolic Blood Pressure
ECU	Equivital Chest Unit
ETT	Endo-Tracheal Tube
HR	Heart Rate
ID	Identification
OPA	Oro-Pharyngeal Airway
O_2 Saturation	Oxygen Saturation
PIV	Peripheral Intravenous
PPE	Personal Protective Equipment
SBP	Systolic Blood Pressure
ST	Skin Thermistors

General CRF Instructions

- Anytime you see: I__I__I:I__I__I → Record the time the participant starts or finishes a task.
- For tasks that have a 'done well' and 'done' grading, If you give a 1 → write in the comments section why you didn't give full points.
- Any decimal places ≥ 0.05 → round up to one decimal place (i.e. 1.85 → 1.9)

Comment Section (Times and Description)
To the right of the checklists is a blank column where the study personnel can describe in their own words any deviation in the actions of the participant from the expected task and note the following: (*See abbreviations in footnote of CRF*).

Footnotes
Throughout the simulation be sure to watch out for the following and record in the comment section.

R = Reminded (Letter, Time and Description)

- Write the letter R in comment section when:
1. Any time the study personnel verbally or physically told the participant what to do (i.e. providing verbal instruction or gesturing the correct step).
2. If the participant is stuck or confused on what to do next and asks for help
N:B. i) remember to check "not done" in the rating scale, and ii) tally the total number at the bottom of the task

P = Blood Pressure-BP, Heart Rate-HR and thermal scale pause (Letter, Time and Description)

- Write the symbol P at the start and stop times of the BP and HR measuring pause
- Record these (BP and HR) values in the observer sheet (if one study personnel) or in the CRF (if two study personnel).
- Record the participants thermal comfort scale reading.
- This will occur every 10 minutes.

B= Breach in PPE (Letter, Time and Description)

- Write the letter B, the time of the event and a quick description of the breach
- Tally the total number of breaches at the bottom of the task.

Minor breach

- There are 7 types of minor breaches under the Participant safety form – Minor Breaches # 159.
- Minor breaches occur anytime the participant's hands are above their nipple line or there is a tear in the PPE OR:

a) Participant attempts to adjust facial PPE
b) Touches or scratches any area around the head.
c) Participant attempts to adjust face mask or face shield OR malposition of the face shield.
d) Tear in coverall, gloves or apron – Should be observed by study personnel or by the participant, the personal protective equipment can be reviewed and examined for rips after the simulation is complete.

e) A fluid (blood) splash on the PPE of the participant but not involving mucous membranes. This splash should be below the level of the nipple line.
- Note the location of the PPE breach.

Major breach

Record the number and describe the type of major breach incident(s) that happened to the participant. These could include the following;
a) Exposure of the broken skin (in this study, exposure of intact bare skin might suffice), mouth, eyes or nose to the patient's body fluids (blood).
b) Contact of the dirty gloves to the inside of the PPE – Observed by study personnel or recorded by participant in the post-simulation questionnaire.
c) Needle stick or other injury- are from study personnel observation and the post-simulation questionnaire.

N = Near-miss Incident (Letter, Time and Description)

- Write the letter N, the time of the event and a quick description of the incident when:
a) Participant doesn't immediately dispose of sharps in the sharps bin (i.e. leaves it on the table next to them).
b) Participant recaps needle.
c) Participant walks with exposed needle, soiled waste (drape, gauze, alcohol wipes or syringe with blood).
- Tally the total number at the bottom of the task.
NB: Walking with blood collection tubes is not a near-miss.

H = Health-concerning Incident (Letter, Time and Description)

From study personnel observation, participant's complaints, physiological measurements or study personnel's discretion (participant loses focus or cannot follow instructions).

- Write the letter H, the time of the event and a quick description of the incident when:
a) The participant surpasses any of the physiological cut offs.
b) Thermal scale rating was greater than 12 or at any point patient feels uncomfortable.
c) The participant asks you more than thrice to read the case scenario or to repeat any information in the scenario.
d) Participant felt unwell and had to leave the simulation.

- Tally the total number at the bottom of the task.

I = Interruption (Letter, Time and Description)

- Participant forgets equipment in green zone, at the work station or at previous patient bed.
- Participant forgets and has to return to previous or early tasks
- Unnecessary repetitive steps or when participant is not sure of what to do next like unnecessary changing of gloves.

Tally: At the end of each task record the total number of recorded Interruptions (I).

Rating Scale

a). Done well: Attracts a score of two points. This is awarded to a participant who satisfactorily performs the task according to the script described under each task item.
N.B: This may not apply to all tasks, because some task items are dichotomous, it is either the participant does it or they do not do it (shaded black boxes in the CRF).

b). Done: Attracts a score of one point. This is awarded to a participant who performs the task but not satisfactorily to the level its defined in the script under each task item except for task items that are dichotomous (i.e. done and not done).

c). Not done: This box is checked when:
a) A participant doesn't do a particular task or skips/misses the task
b) A participant is stuck and asks you for help or asks you what to do next.
- Write an "R" in the comment section indicating that you had to remind the participant at that step. (Note: Reminders can be verbal (i.e. instructions) or non-verbal (i.e. hand gestures or pointing).

d). Not sure: This box is checked when:
a) The study personnel did not observe participant perform a particular task.
b) The study personnel are not sure whether task has been performed.
c) The study personnel did not sufficiently observe participant doing a particular so as to make a clear judgment.

Color Fonts.
Blue – Items in blue mean that this task depends on order. The participant will only receive a checkmark for "Done" if they perform the task in that *particular* order
Orange – Items in orange mean this task can be checked after the simulation is complete

Participant ID- Insert the unique alphanumeric identification of the participant at the top of every page of the CRF, the post simulation questionnaire and the observer sheet.

Observer's Initials- Put the Initials of the person filling in this CRF.

1: Study date- Insert the date in in the following format (DD/MM/YYYY)

2: Participant's Age- Can be found on the pre-simulation questionnaire

3: Record the time of the following measurements:

Green Zone Temperature (°C) (Record to 1 decimal place) - plug device reader into the anal probe taped on the wall, turn it on and record the temperature.
Green Zone Humidity (%) - read from the handheld humidity device
NB; Record the temperature and humidity before the participant starts donning.

4. Did the participant re-watch any of the videos:
- Check any of the videos the participant re-watched.
- Record the number of times the participant watched each of the videos.

- Also keep track of the start time and finish time that the participant takes to watch the videos.

Vitals # 1: Pre-simulation physiological parameters (Baseline).

5. Heart Rate-HR, Systolic Blood Pressure-SBP and Diastolic Blood Pressure-DBP

Technique :

a). Use the ihealth BP cuff (make sure it is charged) and ihealth app (have it downloaded on your mobile phone/gadget).
b). Ensure cuff is paired to the iPhone app via Bluetooth (a blue light will flash).
c). Have participant take a seat.
d). Wrap BPM cuff on participant's non-dominant arm.
e). Depending on blood pressure machine (BPM), connect wireless connection and start measuring the BP.
f). Record both readings of SBP, DBP, HR and the time taken they were taken.

6. Tympanic temperature- (Record to 1 decimal place)

Technique :

a). Have the participant take a seat as you take their tympanic temperature.
b). Pull the pinna (upper part of outer-ear) upward and outward as you insert the tympanic thermometer to get a reading. (For more information, read manual of tympanic thermometer).
c). Do so for both left and right ears.

7. Chest size measurement and Equivital size– Record in centimeters and select the corresponding Equivital size.

- Have the participant stand upright with hands spread out.
- Using the tape measure, wind the tape fairly tightly about the participant's chest at the level of the nipples (males) and just below the breasts (females).

Note: Preferable to demonstrate this procedure and have participants do it on by themselves

➡ *Wet the equivital sensors just prior to attaching them to the participants.*

➡ *Have the participant go into the dressing room and take the unclothed weight of the participant.*

8. Weight: Record the weight in kilograms (Kg) to 1 decimal place.

Technique :

a) Have the participant remove all their clothing to include the undergarments and stand on the scale with their feet apart.
b) Record first weight measurement.
c) Have the participant step off the scale, zero the scale and get them to step back on
d) Record second weight measurement.

9. Height- Record the value in centimetres

Technique :

a). Ensure participant has removed their shoes.

b). Have participant stand against the tape measure that is plastered against the wall.
c). If participant is taller than you, step on a stool.
d). Place a ruler on the participant's head, ensure the ruler is as horizontal as possible then take that reading.

➡ *Attach the BP machine onto the non-dominant arm of participant before they start donning.*
➡ *Check the iPad Equivital app to ensure the Equivital is functioning before the participant starts donning*.

DONNING

10. Record the time the assessor says "begin donning"

11: Inspects PPE to ensure it is of appropriate size, quality and they are no breaches.
Done well: If participant meticulously inspects at least 3 out of the 6 PPE items (i.e. 2 pairs of gloves, coverall, mask, face shield, boots and apron) for appropriate size and any breaches.
Done: If participant briefly inspects less than 3 of the PPE items.
Not done: skips this step or is reminded
Not sure: See description on page 4.

12: Sanitizes hands.
- Done: If participant performs hand washing before he/she starts putting on the PPE.
- Not done: See description on page 4.
- Not sure: See description on page 4.

13: Boots: Removes shoes and puts on rubber boots (correct size).
Done: If participant removes their shoes and puts on the boots before putting on other items of the PPE.
Not Done: If participant puts on other items of the PPE (apart from the first pair of gloves) first before the boots OR if you remind them to put on their boots.

14: Gloves. Puts on the first pair of gloves (nitrile).
Done: If participant puts on first pair of gloves as the next step after sanitizing their hands or after the putting on the boots.
Not done: Skips this step or is reminded.

15: Zips: Puts on coverall and zips up entire fly.
- Done: If participant dons coverall.
- Not done: If participant dons another item of PPE not the coverall OR participant is stuck and requests to be reminded. N.B: Remind them and write a note in the comment section.

16: Cuffs: Slips thumbs into the cuffs.
- Done: If participant slips gloved hands into the cuffs.

- Not done: If participant skips this step and goes to next step OR if they are stuck and ask you to remind them OR if they don another item of PPE before this task OR if they slip only one thumb of either hand into the cuffs.

17: Mask: Puts on face mask-N95 (half-sphere) correctly.

- Done well: If participant puts on face mask-N95 correctly by performing a seal check, places 1 strap above the ears and another strap below the ears.
- Done: If participant puts on face mask-N95 but does not perform seal check OR they don't put the straps above and below the ear OR they have the straps crisscrossing.
- Not done: See description on page 4.

N.B: The mask can be uncomfortable, so some participants would prefer putting it on last but it should always before the face shield.

18: Face Shield: Puts on face shield.

- Done well: If participant puts on the face shield inside the hood.
- Done: If participant puts the face shield outside the hood.
- Not Done: See description on page 4.

19: Hood: Pulls the coverall hood over the head.

- Done well: If participant puts on the hood and ensures the following: face shield is inside hood; no hair or straps are dangling outside the hood and minimal skin as possible around the head is exposed.
- Done: If participant puts on the hood but doesn't ensure any of the following: face shield is inside the hood (no hair or straps are dangling outside the hood and minimal skin as possible around the head is exposed).
- Not done: See description on page 4.

20: Apron : Puts on disposable apron.

- Done: If participant puts on apron.
- Not done: See description on page 4.

21: Gloves: Puts on second pair of long gloves over the coverall cuff.

- Done well: If participant puts on second pair of long nitrile gloves over the coverall and ensures there is no exposed skin or inner pair of gloves.
- Done: If participant puts on second pair of long nitrile gloves but does not ensure: to check whether both gloves are over the coverall; there is no exposed skin or inner pair of gloves.
- Not done: See description on page 4.

22: Self Check: Performs a self-check in the mirror OR asks buddy to help.

- Done well: If participant performs self-check in the mirror and adjusts the PPE items to ensure they are well worn.
- Done: If participant performs check in the mirror but doesn't adjust the PPE items that are not well worn. N.B: Make a note in the comment section what the participant left out.
- Not done: See description on page 4.
- Not sure: See description on page 4.

Record the number of times these events happened; Participant had Interruptions (I), Reminded (R), Breaches (B) and Near miss incidents (N), See description on pages 2 and 3 above. Also record the time when the participant finishes the donning.

Measuring Vitals and roles of study personnel before participant enters the chamber.

Vitals #2: POST-DONNING.
23:HR, SBP and DBP: Requires you to fill in the <u>heart rate, SBP and DBP</u> of the participant under the value column. See detailed description on task item 5 above.

24: Average tympanic temperature: Requires you to fill in the <u>average tympanic temperature</u> of the left and right ears while participant has PPE on. Record the value in degrees Celsius (^{0}C).
Technique :
a). Have the participant take a seat as you take their tympanic temperature.
b). Remove/adjust the communication system from participant, to slightly expose ears on both sides, pull hood slightly to the side.
c). Pull the pinna (upper part of the outer ear) upward and outward as you insert the tympanic thermometer to get a reading.
d). Do so for both ears and record each reading. Record the time the average tympanic temperature is taken.
e). Place back the hood and the communication system.

<u>Post-Donning checklist.</u>
General instructions.

- These parameters are taken while the participant is in PPE and in the green zone ("zone with reduced risk of infection transmission").
- Ensure the different equipment is functional and the batteries are charged as the participant was donning.
- Speaker headset: Equip participant with a microphone and speaker system and test for two-way communication.
- Record the participant identification number-ID #: Write the unique ID # and time on the front and back of his/her coverall.
- Turn off donning camera.
- Plug in ACR to get the temperature of the chamber.
- Record the ETU chamber temperature (^{0}C), humidity (%) and time just before the participant enters chamber.

Items (23- 24) do not attract a score for the participant, it is the role of the study personnel to perform them.

⟶ Ready? Ask the participant whether they are ready to enter the simulation room.
Note; Do not remind them about taking the equipment into the red zone unless they have forgotten.

Red Zone Patient Care.

General instructions: (In addition to those in the green zone)

- All patient scenario cases will be done in the red zone.
- The participants can only leave the red zone after they have finished the simulation process or whenever they choose to terminate their participation OR when they surpass their individual safety cut-offs.

TASK 1: Peripheral Intravenous Insertion (PIV)

25: Enters ETU with task equipment.

- Done: If participant enters ETU with the equipment required to perform the tasks.
- Not done: If participant enters the ETU without the task equipment OR if you remind them to take task equipment into the ETU. N.B: Make a note in the comment section if you reminded-(R) them or helped bring in their task equipment.

N.B: Remember to record the time they enter the chamber.

26: Time the assessor starts reading out scenario.

Record the time off the digital clock as the assessor begins reading the case scenario to the participant.

27. Did the participant ask the assessor to repeat anything?

Make a note in the comment section, if participant wants you to repeat the scenario or asks for any parts of the script to be repeated (i.e. scenario was read 2 times, participant wanted vitals repeated, etc.)

Check Y for yes, N for No. If yes, record the number of times that the case scenario was read out to the participant. N.B: This should prompt you to observe for any incoherence to instruction by participant or early warning signs for heat stress and exhaustion.

28: Time the assessor finishes reading out scenario to participant.

Record the time when the assessor finishes reading case script to the participant. If participant requests for the case to be read out again, then record the time after the final read out.

29: Introduces self to the patient.

- Done: If participant introduces self to patient OR makes an effort to explain procedure OR interacts with the patient.
- Not done: If participant does not introduce self to patient or explain procedure to patient.
- Not sure: see description on page 4.

30: Sharps Bin: Places sharps bin within arm’s reach.

- Done: If participant places sharps bin within arms-reach before starting the procedure.
- Not done: If participant does not place sharps bin within arms-reach OR Places it behind themselves (beyond their field of vision).

31: Sanitizes gloved hands.

See detailed description under task item 12.

N.B: Check done if participant does this step before touching patient, check not done if participant proceeds to touch patient without sanitizing their hands.

32: Tourniquet: Applies tourniquet and rechecks selected vein.

- Done: If participant applies tourniquet on patients arm at a site above the selected vein and prior to insertion of the needle.
- Not done: If participant skips this step OR participant does not apply the tourniquet on the patients arm OR if participant is stuck and requests you to remind them.

NB: Record the time.

33: Finds Vein: Locates a suitable vein through inspection and palpation.

- Done: If participant inspects by looking at different sites on the arm for suitable veins OR palpates the arm to find a good vein OR utilizes the picture of the different veins on the arm.
- Not done: If participant does not inspect or palpate arm for suitable vein OR if participant skips this step and performs other steps OR if participant is stuck and requests you to remind (R) them.
- Not sure: see description on page 4.

34: Sanitizes procedural site.

- Done well: If participant uses alcohol wipes to clean selected surface, does it in a proper fashion (i.e.; starts to clean the intended site from inwards to outwards in a circular manner OR in a vertical way starting from the site then outwards) and allows site to dry.
- Done: If participant uses alcohol wipes to clean area, but cleans site hurriedly in a haphazard fashion and doesn't allow site to dry.
- Not done: see description on page 4.
- Not sure: see description on page 4.

35: Inserts needle into vein.

- Done well: If participant successfully finds the vein on 1st or 2nd attempts. Success being defined as having a functional cannula in the vein.
- Done: If participant attempts more than 2 times.
- Not done: see description on page 4.
- Not sure: see description on page 4.

NB: Record the time corresponding to any new attempt.

36-37: Confirms needle is in the vein: 1. By noticing a backflow of blood in the catheter (informs the study personnel that they can see the backflow of blood) OR 2. By aspirating blood through the cannula OR 3. Line is flushed with sterile water.

- Done: If participant does any one of the three above. aspirates blood by withdrawing the plunger of the syringe
- Not done: See description on page 4.
- Not sure: See description on page 4.

a) How many needle pricks did participant do before they were successful? Tally:

This step requires you to tally how many needle pricks the participant did before they were successful.

b) How many cannula did participant use before they were successful? Tally:

This step requires you to tally how many cannula the participant used before they were successful.

38: Releases Tourniquet.

- Done well: If participant releases tourniquet immediately after they have confirmed IV access has been established.
- Done: If participant releases tourniquet after proceeding to next step in the sequence (that is after disposing off the sharps needle into the sharps bin).
- Not done: If participant does not remove the tourniquet at all OR if participant is stuck and asks to be reminded.
- Not sure: See description on page 4.

NB: Record the time when participant removes the tourniquet.

39: Disposes off sharps in sharps bin.

- Done – immediately disposes of sharps in sharps bin
- Not Done – recapped the needle, or did not dispose it off immediately, or walked with exposed needle or left needle exposed – record in comments as Near-miss incident section. You could check after the simulation what participant did.

40: Secures peripheral IV cannula.

- Done: If participant secures peripheral IV cannula with tape or tegaderm.
- Not done: See description on page 4.

41: Samples; Obtains bloodwork.

- Done well: If participant obtains blood samples in both blood tubes.
- Done: If participant obtains blood samples in one blood tube.
- Not done: See description on page 4.

N.B: Remember to record the time when the blood samples were collected. Check after the simulation to confirm how participant did it.

42: IV: Initiates IV fluid therapy.

- Done well: If participant initiates IV fluid therapy by connecting fluid bag to catheter lumen and ensures that it is flowing (opens clamp on primary IV tubing to confirm flow of fluid OR closes clamp and uses empty syringe to aspirate cannula and then flushes the cannula with sterile water) OR demonstrates any of these attempts.
- Done: If participant initiates IV fluid therapy by connecting fluid bag but does not check to ensure that fluid is flowing.
- Not Done: See description on page 4.

N.B: Also record the time when the participant has completed this task. Check after the simulation or video clip to confirm whether fluid is running or if you were not sure.

43: End Cap: Applies end cap to lumen.

- Done: If participant applies end cap to the lumen.
- Not Done: See description on page 4.
- Not sure: See description on page 4.

44: Wipes blood tubes with disposable paper towel and alcohol wipes.

- Done well: If participant uses disposable paper towel to wipe both blood tubes and disposes paper towel into the appropriate bin.
- Done: If participant uses paper towel to wipe one instead of both blood tubes and does not dispose off the paper towel OR any other variant to 'done well' above.
- Not Done: If participant does not wipe the blood tubes OR If participant skips this step.
- Not sure: If you did not observe the participant doing this step so as to make a clear judgment OR you are not sure if participant performed this task.

45: Wraps blood tubes with napkins.

Done: If participant wraps both tubes with napkins before placing them into the secondary tubes.
Not Done: If participant skips this step OR participant is stuck and asks to be reminded.

46: Sanitizes gloved hands after wrapping primary blood tubes and before placing them into the secondary tubes.

- Done: If participant sanitizes their hands in between wrapping/disinfecting the primary tubes and placing them in the secondary tubes.
- Not done: If participant does skip this step.

47: Secondary Tubes: Places blood tubes into secondary tubes.

- Done well: If participant places the correct tubes in their respective labelled secondary tubes and tightly closes the them.
- Done: If participant places the wrong blood tubes into the secondary tubes.
- Not done: If participant does not place blood tubes into the secondary tubes OR if they skip this step and proceed to another step and perform this later OR If participant is stuck and asks you to help them.

N.B: Check after the simulation or watch the video clip to confirm how participant did it.

48: Disinfects any blood from the site.

- Done well: If participant disinfects all of the site(s) that could have been contaminated with the blood.
- Done: If participant does not disinfect all of the site(s) that could have been contaminated with blood.
- Not done: See description on page 4.
- Not applicable (N/A): If the site was clean and there was no visible blood contamination to require disinfecting.
- Not sure: See description on page 4.

N.B: Check after the simulation or video clip to confirm how participant did it.

49: Waste: Properly segregates and disposes waste (soiled gauze, syringes, etc.)

- Done well: If participant properly segregates and disposes off the waste (i.e. sharps into the sharps bin/yellow bin), non-sharps non-infectious waste i.e. paper, plastic wrapping, obturator, drug preparations, gown and drape (because of size) into the black bin, blood/fluid stained gauze/cotton/paper/syringes/gloves into the red bin).

- Done: If participants discards some of the waste but leaves some on the patient's bed OR segregates them inappropriately.
- Not Done: See page 4.
- Not sure: See page 4.

50: Sanitizes gloved hands before putting on a new pair of gloves.

See detailed description on task item 12.

This step should be done before 51 and only then can it be graded as done, otherwise, it is counted as a not done.

51: Changes: Removes outer pair of gloves and puts on a new pair of gloves before moving to the next patient.

- Done well: If participant removes outer pair of gloves making sure they maintain (outside to outside and inside to inside technique). Then puts on second pair of long nitrile gloves over the coverall cuffs and ensures there is no exposed skin or inner pair of gloves.
- Done: If participant removes the outer pair of gloves but they do not maintain (outside to outside and inside to inside) technique OR If participant puts on second pair of long nitrile gloves but does not ensure: to check whether both gloves are over the coverall cuff; there is no exposed skin or inner pair of gloves. If skin is exposed mark as B in comments and record Breach in the patient safety form.
- Not done: See description on page 4.
- Not sure: See description on page 4.

Record the time when the participant has finished the task.

See definitions of task completion in Case scenario scripts.

1. The participant reports they have finished the scenario

2. The participant completes all the required tasks in the CRF (but doesn't verbally report that they are done)

3. The participant is unable to do the task and wants to move on.

Record the number of times these events happened; Participant Interruptions (I), Reminded (R), P (SBP, DBP, HR and Thermal Scale), Breaches (B), and Near miss incidents (I), See description on pages 2 and 3 above.

Task 2: Mid-Line Catheter insertion (MLC).

52: Time the assessor starts reading out scenario

Record the time off the digital clock as the assessor begins reading the case scenario to the participant.

53: Did the participant ask the assessor to repeat anything?

- See detailed description under item 27 above.

54: Time the assessor finishes reading out scenario to participant

- See detailed description under item 28 above.

55: Introduces self to the patient.

- Done: If participant introduces self to patient OR makes an effort to explain procedure OR interacts with the patient.
- Not done: See description on page 4.
- Not sure: See description on page 4.

56: Sharps Bin: Places sharps bin within arms' reach.

See detailed description under task item 30.

57: Sanitizes hands immediately prior to procedure.

See detailed description under task item 14.
This step should be done before 58 and only then can it be graded as done. otherwise, it is counted as not done.

58: Sanitizes procedural site.

See detailed description under task item 34.
This step should be done before putting on Drape and only then can it be graded as done, otherwise, it is counted as a not done.

59: Sterile Field: Sets up sterile field in a sterile way and opens the gown, gauze, adhesive tape, fenestrated drape, syringe, 4-way stopcock mid-line kit and tegaderm.

- Done well: If participant sets up field in a sterile way by opening the gown, gauze, adhesive tape, fenestrated drape, syringe and mid-line catheter. Ensures that they do not touch the inside of any of the equipment while unwrapping them from their packaging.
- Done: If participant sets up field but doesn't not maintain sterility while setting up the sterile field (i.e. they touch the inside of the equipment while unwrapping them from their packaging) OR participant does not open all the equipment.
- Not done: See detailed description on page 4.
- Not sure: See detailed description on page 4.

Record the time duration they take to set up this. Note any interruptions

60: Sanitizes hands immediately prior to procedure.

See detailed description on task item 12.
This step should be done before 63 (before putting sterile gloves) and only then can it be graded as done, otherwise, it is counted as a not done.

61: Removes: Removes outer pair of gloves.

- Done well: If participant removes outer pair of gloves making sure they maintain (outside to outside and inside to inside) technique.
- Done: If participant removes the outer pair of gloves but they do not maintain (outside to outside and inside to inside) technique.
- Not done: See detailed description on page 4.
- Not sure: See detailed description on page 4.

62: Gown: Puts on the sterile gown.

- Done well: If participant puts on sterile gown making sure they do not touch their face or disrupt the integrity of other parts of the PPE such as putting mask/hood/face shield out of position AND ties the straps at the side.
- Done: If participant puts on sterile gown but they touch their face or disrupt the integrity of other parts of the PPE like putting mask/hood/face shield out of position OR doesn't tie the straps. N.B: Note the first part of the 'done' definition as a minor breach (B)
- Not done: See detailed description on page 4.
- Not sure: See detailed description on page 4.

63: Gloves: Puts on the sterile gloves.

- Done well: If participant puts on new pair of sterile gloves making sure they maintain (outside to outside and inside to inside) technique. Ensures sterile pair of gloves covers the coverall and that there is no exposed skin or inner pair of gloves.
- Done: If participant puts on new pair of sterile gloves but they do not maintain (outside to outside and inside to inside) technique OR If participant puts on sterile pair of gloves but does not ensure: to check whether both gloves are over the Gown; and that there is no exposed skin or inner pair of gloves.
- Not done: See detailed description on page 4.
- Not sure: See detailed description on page 4.

64: Drape: Uses sterile technique to drape patient: Follows body diagram on the drape.

- Done well: If participant uses sterile technique to drape the patient i.e. Follows body diagram on the drape.
- Done: If participant drapes the patient but does not follow body diagram on the drape and has to re-adjust it.
- Not done: See detailed description on page 4.
- Not sure: See detailed description on page 4.

NB: Record the times corresponding to when the participant starts and finishes placing the drape.

65: Sanitizes identified procedural site.

See detailed description under task item 34.
This step should be done before 67 (lidocaine) and after 64 (Drape) and only then can it be graded as done well/done. otherwise, it is counted as a not done.

66: Flush: Checks functionality of mid-line catheter by flushing the lumens and clamping them.

Done well: If participant checks functionality of the mid-line catheter by flushing BOTH lumens of the catheter with normal saline/sterile water.
Done: If participant checks functionality of the mid-line catheter by flushing only one lumen of the catheter.
Not done: See detailed description on page 4.
Not sure: See detailed description on page 4.

67: Anesthesia: Safely injects lidocaine into the skin over the location of the palpated vein. Remind participant to say out loud whatever they are doing.
Done well: If participant safely inserts the needle (plane of needle should be at an acute angle to the skin surface) into the skin AND ensures the needle is not in the vein by slightly withdrawing the plunger.
Done: If participant inserts the needle (while plane of needle is not an acute angle to the skin surface) OR does not ensure whether needle is in the vein by slightly withdrawing plunger.
Not done: See detailed description on page 4.
Not sure: See detailed description on page 4.

68: Disposes of sharps immediately in sharps bin.
See description under item 39.

69: Inserts needle into the vein.
See description under item 35.

70: Aspirates: Confirms needle is in vein by aspirating blood through the cannula.
See description under item 36.

71: How many needle pricks did participant do before they were successful? Tally:
This step requires you to tally how many needle pricks participant did before they were successful.

72: Guide Wire: Advances the guide wire through the needle into the vein as the needle is being removed and leaves the guide wire in the vein.
- Done: If participant advances guide wire through the needle into the vein as the needle is being removed and leaves the guide wire in the vein.
- Not done: See detailed description on page 4.
- Not sure: See detailed description on page 4.

73: Removes Needle.
- Done: If participant removes the needle
- Not Done: See detailed description on page 4.
- Not sure: See detailed description on page 4.

74: Disposes of sharps immediately in sharps bin.
- See description under item 39.

75: Introducer and Dilator: Threads the introducer and the dilator over the wire into skin and vein.
- Done: If participant threads the introducer and the dilator over the wire into the skin and vein.
- Not done: See detailed description on page 4.
- Not sure: See detailed description on page 4.

76: Removes the guide wire.
- Done well: If participant removes the guide wire while placing it back in the straw OR discards it into the sharps container.
- Done: If participant removes the guide wire and places it on the tray.
- Not done: See detailed description on page 4.
- Not sure: See detailed description on page 4.

77: Removes the dilator but leaves the introducer in place.
- Done: If participant removes the dilator but leaves the introducer in place
- Not done: See detailed description on page 4.
- Not sure: See detailed description on page 4.

78: Catheter and Introducer: Inserts the mid-line catheter and obturator into the introducer and ensures entire catheter length is in vein.
- Done well: If participant Inserts the mid-line catheter and obturator into the introducer and ensures entire catheter length is in vein.
- Done: If participant does not insert entire length of the mid-line catheter into the vein.
- Not done: See detailed description on page 4.
- Not sure: See detailed description on page 4.

Record the time participant finishes inserting Catheter.

79: Removes the peel away introducer.
- Done well: If participant effortlessly (1-2 times) removes the peel away introducer, by pulling outward and apart on the two wings of the sheath.
- Done: If participant struggles (tries more than twice) to remove the peel away introducer.
- Not done: See detailed description on page 4.
- Not sure: See detailed description on page 4.

80: Removes Obturator.
- Done: If participant removes the obturator.
- Not done: If participant does not remove the obturator OR participant forgets to remove the obturator.
- Not sure: See detailed description on page 4.

81: Aspirates blood from each lumen (to avoid air embolism and ensure intravascular placement).
- Done well: If the participant aspirates blood from each lumen of the mid-line catheter and also flushes them to check for functionality.
- Done: If the participant aspirates blood from only one lumen OR does not flush the lumen of the catheters.
- Not done: See detailed description on page 4.
- Not sure: See detailed description on page 4.

82: Stabilization device: Secures the catheter.
- Done: If participant secures peripheral IV cannula with tape or tegaderm.
- Not done: See description on page 4.

Check after simulation to confirm if participant used it, if you are not sure.

83: Samples: Obtains bloodwork.

- See description under item 41.

N.B: Remember to record the time when the blood samples were collected.

84: 4-way stopcock:

Done well: Applies a 4-way stopcock to the lumen of the catheter and tests its flow.
Done: If participant applies the 4-way stopcock but does not test its' flow.
Not done: If participant doesn't apply the 4- way valve.

85: Initiates IV fluid therapy.

- See description under item 42.

N.B: Record the time when the participant has completed this task.

86: End Cap: Applies end cap to one of the lumens of the catheter.

- Done: If participant applies end cap to the lumen of the catheter.
- Not Done: If participant doesn't not apply end cap to the lumen OR if participant skips this step.
- Not sure: See detailed description on page 4.

87: Wipes blood tubes with disposable paper towel.

- See description under item 44.

88: Wraps blood tubes with napkins.

- See description under item 45.

89: Sanitizes gloved hands.

See detailed description under task item 46.
This step should be done before 91 and only then can it be graded as done, otherwise, it is counted as a not done.

90: Secondary Tubes: Places blood tubes into secondary tubes.

- See description under item 47.

91: Secondary Tubes: Disinfects the secondary tubes after tightly closing them.

See detailed description under task item.

92: Disinfects any blood from the site.

- See description under item 48.

93: Dressing: Applies a transparent film dressing over the insertion site.

- Done: If participant applies a transparent film dressing over the insertion site.
- Not done: If participant proceeds to another task before completing this one OR participant is stuck on what to do next and asks for help. N.B: Remind participant and make a note in the comment section.

94: Drape: Removes drape from patient and disposes it safely.
- Done well: If participant removes drape from patient in a way that they don't contaminate themselves (In reverse order as to how drape was put).
- Done: If participant removes drape from patient in a way that they put themselves at risk of contamination (i.e. they don't follow the reverse order as to how drape was put).*Watch out for any breaches-B*
- Not done: See detailed description on page 4.

Record the time.

95: Sanitizes gloved hands.

See detailed description under task item 12.

This step should be done before 96 (removal of sterile gloves) or after removal of the drape, and only then can it be graded as done. otherwise, it is counted as a not done.

96: Removes sterile gloves.
- See description under item 51.

97: Sanitizes gloved hands after removal of sterile gloves.

See detailed description under task item 12.

This step should be done before 98 and only then can it be graded as done. otherwise, it is counted as a not done.

98: Removes sterile gown taking care not to contaminate self.
- Done well: If participant removes sterile gown making sure they do not touch their face or disrupt the integrity of other parts of the PPE like putting mask/hood/face shield out of position.
- Done: If participant removes sterile gown but they touch their face or disrupt the integrity of other parts of the PPE like putting mask/hood/face shield out of position.
- Not done: See detailed description on page 4.
- Not sure: See detailed description on page 4.

99: Waste: Properly segregates and disposes waste (soiled gauze, syringes, etc.)
- See description under task item 49.

100: Sanitizes gloved hands.

See detailed description under task item 12.

This step should be done before 101 or after 99 and only then can it be graded as done, otherwise, it is counted as a not done.

101: Changes: Puts on a new pair of gloves before moving to the next patient.
- See description under task item 51.

Record the time when the participant has finished the task.

See description in box on page 13.

Record the number of times these events happened; Participant Interruptions (I), Reminded (R), P (SBP, DBP, HR and Thermal Scale), Breaches (B), and Near miss incidents (I), See description on pages 2 and 3 above.

Task 3: Intubation and Mechanical Ventilation (ETI).

102: Time the assessor starts reading out scenario .
- See detailed description under item 26 above.

103: Did the participant ask the assessor to repeat anything?
- See detailed description under item 27 above.

104: Time the assessor finishes reading out scenario to participant.
- See detailed description under item 28.

105: Introduces self to the patient.
- See detailed description under item 29.

106: Sanitizes hands before touching patient.
See detailed description under task item 12.
This step should be done before 107 and only then can it be graded as done. Otherwise, it is counted as a not done

107: Clears the airway: Removes blockage before the airway and suctions the oral cavity.
- Done: If participant first inspects the oral cavity, then removes the blockage (paper in the mouth).
- Not Done: If participant does not inspect the oral cavity to remove the blockage but then proceeds to do the suctioning of the oral cavity or places airway mask on patient.
- Not sure: see description on page 4.

108: Clears the airway: suctions the oral cavity.
- Done: If participant first inspects the oral cavity, then suctions it before applying the airway mask.
- Not Done: If participant does not inspect the oral cavity to suction but then proceeds to place airway mask on patient.
- Not sure: see description on page 4.

109: Airway Mask: Begins to pre-oxygenate patient with airway mask.
- Done well: If participant places appropriate sized airway mask on patient and ensures that a proper seal about the mouth is made then begins to pre-oxygenate patient.
- Done: If participant places the wrong sized airway mask on patient and then begins to pre-oxygenate patient.
- Not done: See description on page 4.
- Not sure: see description on page 4.
Record the time when the participant conducts this.

110: ETT Prep: Ensures cuff on the endotracheal tube (ETT) can be inflated and deflated and that there are no air-leaks.

- Done well: If participant ensures cuff on the endotracheal tube (ETT) can be inflated and deflated and that there are no air-leaks by using a 10 ml empty syringe.
- Done: If participant either deflates or inflates the cuff, but doesn't do both actions.
- Not done: See description on page 4.
- Not sure: See description on page 4.

N.B: This step can be done anytime as long as it comes before 116.

111: Deflates the cuff and then inserts the stylet.

- Done well: If participant deflates the cuff on the ETT by using an empty 10 ml syringe and then inserts the stylet.
- Done: If participant does not deflate the cuff on the ETT but inserts the stylet.
- Not done: See description on page 4.
- Not sure: See description on page 4.

N.B: This step can be done anytime as long as it comes before 116.

112: Checks laryngoscope function.

- Done: If participant checks the laryngoscope is functional (light works)
- Not done: See description on page 4.
- Not sure: See description on page 4.

N.B: This step can be done anytime as long as it comes before 116.

113: Aspirates to ensure IV access is working well.

- See description under task item 42.

N.B: Record the time when the participant has completed this task.

114: Medications: Administers intubation-related medications like sedatives and muscle relaxants.

- Done well: If participant says out loud that he/she is administering both the sedative and a muscle relaxant or check if medication syringes are still filled after the simulation. OR gives at least 2 of the 3 medications (Propofol, Rocuronium and Ketamine).
- Done: If participant says out loud that he/she is administering only one of following drugs; Propofol, Rocuronium and Ketamine.
- Not done: See description on page 4.
- Not sure: See description on page 4.

N.B: Record the time when the participant has completed this task

115: Removes airway mask.

- Done: If participant removes airway.
- Not done: See description on page 4.
- Not sure: See description on page 4.

N.B: Record the time when the participant has completed this task

116: OPA: Correctly inserts Oro-pharyngeal airway (OPA).
- Done well: If participant removes airway and uses correct size of OPA (by measuring from the corner of the mouth to the angle of the mandible) and uses correct technique to inserts OPA (i.e. OPA into the oral cavity with the tip of the OPA curve facing the hard palate till the posterior wall of the pharynx then rotates it about 180° to proper position) OR simply uses a yellow or green colored OPA.
- Done: If participant removes airway and either uses a wrong size of an OPA i.e. uses a different color of an OPA other than green or yellow.
- Not done: See description on page 4.
- Not sure: See description on page 4

117: Ventilates patient with a Bag Valve Mask-BVM
- Done: Ventilates patient with a Bag Valve Mask
- Not done: See description on page 4.
- Not sure: See description on page 4.

118: Inserts Laryngoscope: Stands behind the patient and tilts patient's head
Done well: If participant while standing behind patient, correctly inserts laryngoscope (technique: With the non-dominant hand inserts the curved blade of the laryngoscope into the mouth, following the midline till the tip of the blade reaches the vallecular, then lifts the laryngoscope upwards and away to expose the vocal cords and laryngeal opening) and tilts patients head. OR Participant is successful in 1 or 2 attempts.
Done: If participant inserts laryngoscope but does not do one or both of the techniques well i.e. if participant appears to struggle (tries more than twice) to complete this step.
Not Done: See description on page 4.
Not Sure: See Description on page 4.

119: Inserts ETT.
- Done well: If participant inserts ETT using correct technique (with the other hand, inserts the ETT directly between the cords towards the trachea until the cuffs passes through the vocal cords) OR participant is successful in 1 or 2 attempts.
- Done: If participant inserts ETT but appears to struggle (tries more than twice) to complete this step.
- Not Done: See description on page 4.
- Not Sure: See Description on page 4.

Record the times for each of the attempts performed.
N.B: Second option-To comfortably check this item (done well OR done on this step) wait till participant has done step 122.

120: Inflates the cuff of the ETT with about 10-15 ml of air.
- Done: If participant inflates the cuff of the ETT with about 10-15 ml of air.
- Not done: See description on page 4 .
- Not sure: See description on page 4.

N.B: This can be confirmed after the simulation.

121: Capnometer/Colorimeter: Inserts an end-tidal CO_2 detector.
- Done: If participant inserts an end-tidal CO_2 detector on the tubing connected to the ETT.

- Not done: See description on page 4.
- Not sure: See description on page 4.

N.B: We could also confirm this after the simulation.

122: Chest Expansion: Confirms correct ETT placement by ventilating with bag-valve apparatus to see symmetrical chest wall expansion.

- Done well: If participant confirms correct ETT placement by ventilating with bag-valve apparatus to see symmetrical chest wall expansion on the first or second attempt.
- Done: If participant attempts more than twice to confirm correct ETT placement by ventilating with bag-valve apparatus to observe symmetrical chest wall expansion OR If participant's ventilation produces asymmetrical chest expansion.
- Not done: See description on page 4.
- Not sure: See description on page 4.

N.B: Record the time for each attempt.

123: How many ETT insertion attempts did the participant perform being successful.

Tally the number of attempts the participant performed before being successful and scribble done the total number.

124: Tapes the ETT to the cheek of patient.

- Done well: If participant uses correct length of tape to secure the ETT on both cheeks of the patient.
- Done: If participant uses a short tape of length to secure the ETT on both cheeks of the patient OR If participant struggles with the tape as the sticky side of the tape gets entangled in the participant's gloves.
- Not done: See description on page 4.
- Not sure: See description on page 4.

125: Connects tube to the ventilator.

- Done: If participant connects tube to the ventilator and turns it on.
- Not done: See description on page 4.
- Not sure: See description on page 4.

126: Turns Ventilator on.

- Done: If participant turns on the ventilator.
- Not done: See description on page 4.

127: Waste: Properly segregates and disposes waste (soiled gauze, syringes, equipment used. etc.)

- See description under task item 49.

128: Sanitizes gloved hands before changing to new pair of gloves.

- See detailed description on task item 12.

This step should be done before exiting the chamber and only then can it be graded as done. otherwise, it is counted as a not done.

129: Changes: Puts on a new pair of gloves before moving to the next patient.
- See description under task item 51

Record the time when the participant has finished the task and leaves the chamber.
See description on page 13.

Record the number of times these events happened; Participant Interruptions (I), Reminded, P (SBP, DBP, HR and Thermal Scale), Breaches (B), and Near miss incidents (I), See description on pages 2 and 3 above.

Doffing Checklist.

Introduction.

The general instructions are quite similar to what has been described under the red zone above.

Specific instructions to the doffing process.
130: Record the time the participant exits the ETU.

131: Boots: Upon exiting the simulation room, disinfects boots by stepping in a bucket of disinfectant.
- Done: If participant upon exiting the simulation room, disinfects boots by stepping in the bucket containing disinfectant.
- Not done: See description on page 4.
- Not sure: See description on page 4.

132: Drops blood samples that are within a secondary container into a bucket containing disinfectant.
- Done: If participant carefully drops all blood samples in secondary containers into a bucket containing a disinfectant at the boundary of the green and red zones.
- Not done: See description on page 4.
- Not sure: See description on page 4.

133: Sanitizes gloved hands before taking off Apron.
See detailed description on task item 12.

134: Apron: Tears the disposable apron at the neck or the front and rolls it down without touching the front area. Rips it from the sides and then rolls the apron forward.
- Done well: If participant tears the disposable apron at the neck, rolls it down without touching the front area and then with both hands rips it from the sides and then rolls the apron forward.
- Done: If participant removes the apron but does not follow the steps in done well above.
- Not done: See description on page 4.
- Not sure: See description on page 4.

135: Sanitizes gloved hands after removing apron.
See detailed description on task item 12.

136: Unzips: Correctly unzips coverall

- Done well: If participant correctly unzips coverall (In front of a mirror, traces for the zipper on the fly by palpating for their navel, then moves to the top as one hand leads the other and unzips taking care not to touch the inside scrubs).
- Done: If participant unzips coverall incorrectly (does not utilize mirror OR traces for zipper on the fly wrongly OR while unzipping touches the inside scrubs). N.B: Consider this a breach.
- Not done: See description on page 4.
- Not sure: See description on page 4.

137: Hood: With both hands, holds the back of the hood, pulls it upwards and backwards.

- Done well: If participant uses both hands simultaneously to hold the back of the hood, then pulls it upwards and backwards to remove hood from the head, making sure they do not touch their face or disrupt the integrity of other parts of the PPE like putting mask/face shield out of position.
- Done: If participant removes hood but does not follow the technique above and touches their face or disrupts the integrity of other parts of the PPE like putting mask/face shield out of position. N.B: Consider this a breach (B)
- Not done: See description on page 4.
- Not sure: See description on page 4.

138: Coverall - Shoulders: Frees shoulders by touching outside of coverall at nipple line level.

- Done well: If participant frees shoulders by touching outside of the coverall at about the nipple line level and above, then shrugs shoulders ensuring that: he/she does not touch the inside of the coverall or scrubs; does not disrupt the integrity of other parts of the PPE like putting mask/face shield out of position.
- Done: If participant frees shoulders but does not follow technique and touches the inside of the PPE or scrubs OR disrupts the integrity of other parts of the PPE like putting mask/face shield out of position. N.B: Consider this a breach (B).
- Not done: See description on page 4.
- Not sure: See description on page 4.

139: Outer pair of gloves: Removes outer pair of gloves while pulling arms out of the sleeves of the coverall (see technique).

- Done well: If participant removes outer pair of gloves while pulling arms out of the sleeves of the coverall. <u>Technique</u>: Ensures that they does not touch the inside of the coverall with the outer surface of the outer pair of gloves, the outer pair of gloves come off as the hands pull through the coverall from the inside.
- Done: If participant removes outer gloves but does not follow the technique above.
- Not done: See description on page 4.
- Not sure: See description on page 4.

140: Using inner gloves, rolls coverall down from inside down to the boots. Uses one boot to step at heel of other boot so as to pull off coverall and vice versa.

- Done well: If participant uses inner gloves to roll coverall down while touching the inside of the coverall, to bring coverall down to the boots OR uses one boot to step at heel of the other boot to pull off coverall and vice versa.

- Done: If participant performs this task but touches the outside of the coverall. N.B: Consider this a breach (B).
- Not done: See description on page 4.
- Not sure: See description on page 4.

141: Steps away from coverall, picks it up and disposes it safely.

- Done well: If participant steps away from coverall and picks it up carefully (holding the inside of the coverall with both hands) and disposes it.
- Done: If performs this task but does not safely pick up the coverall (i.e. touches the outside of the coverall as they pick it up).NB: Look out for a breach.
- Not done: See description on page 4.
- Not sure: See description on page 4.

142: Sanitizes gloved hands after removing coverall.

See detailed description on task item 12.

143: Shield: Removes face shield.

- Done well: If participant with eyes closed, removes face shield by slightly bending head forward and pulling strap from behind and disposes it safely.
- Done: If participant removes face shield but does not follow the technique above (i.e. they do not close their eyes as they remove the face shield or they accidently touch their face).
- Not done: See description on page 4.
- Not sure: See description on page 4.

144: Sanitizes gloved hands after removing face shield.

See detailed description on task item 12.

145: Mask: Removes Mask.

- Done well: If participant removes face mask with eyes closed, slightly bending head forward, removes bottom string first and leaves it hanging in front of the face and then removes the top string starting from the back of head and disposes face mask safely.
- Done: If participant removes face mask but does not follow technique above (i.e. they do not close their eyes as they remove the face mask OR they accidently touch their face-consider this a breach).
- Not done: See description on page 4.
- Not sure: See description on page 4.

146: Sanitizes gloved hands after removing face shield.

See detailed description on task item 12.

147: Hair Cover: Removes hair cover by reaching at the back of the head.

- Done well: If participant removes hair-cover by slightly bending head backward and then reaches for the hair cover and disposes it.
- Done: If participant removes hair-cover but does not follow technique above.
- Not done: See description on page 4.
- Not sure: See description on page 4.

- Not applicable (N/A): If participant did not use the hair-cover.
NB: This item 147 does not count in the redcap, since not all participants will use this step.

148: Sanitizes gloved hands after removing hair cover.
See detailed description under task item 12.
NB: This item 148 does not count in the redcap, since not all participants will use this step

149: Gloves: Removes inner pair of gloves taking care not to contaminate self.
- Done well: If participant removes outer pair of gloves making sure they maintain (outside to outside and inside to inside technique).
- Done: If participant removes the outer pair of gloves but they do not maintain (outside to outside and inside to inside) technique.
- Not done: See description on page 4.
- Not sure: See description on page 4.

150: Boots: Disinfects boots by stepping in a bucket of disinfectant.
- Done: If participant before exiting the red zone, disinfects boots by stepping in bucket containing disinfectant.
- Not done: See description on page 4.
- Not sure: See description on page 4.

151: Hands: Steps in the green/clean zone and washes hands with soap and water.
See detailed description under task item 12.

Record the time when the participant has finished the task.<br>See description in box on page 13.

Record the number of times these events happened; Interruptions (I), Reminded (R), Breaches (B) and Near miss incidents (N) and Health-concerning incidents (H), See description on pages 2 and 3 above.

VITALS # 3 - POST DOFFING.

General instructions:

- These parameters are taken while the participant has finished doffing PPE and in the green zone (“zone with reduced risk of infection transmission”).
- Ensure the different equipment is functional and the batteries are charged as the participant was in the red zone.
- These items (152 to 153) do not attract a score for the participant.

152: HR, SBP and DBP:
See detailed description under item 5.

153: Average tympanic temperature:
See detailed description under item 6.

Change Room

154: Weight:

-See description under item 8.

VITALS # 4: POST-SIMULATION

These parameters are taken at least 10-15 minutes after item 154 (after participant's post doffing vitals have been taken).

- Items 152 and 153 are similar to items 155 and 156 respectively and they don't attract scores for the participant.

GREEN ZONE # 2.

157: Temperature and Humidity of the green zone: This requires you to fill in the temperature and humidity of the green zone after participant has exited red zone. Record the time when these values were taken.

PARTICIPANT SAFETY

PERSONAL PROTECTIVE EQUIPMENT (PPE)

This form covers the safety parameters of the simulation study. Some of these items can be re-examined by watching the video clips.

Section 158-161 is about breaches in PPE. A breach, in the most basic terms, is any deviation in PPE i.e. tears, rips, malfunction or the way it is worn (i.e. items not positioned properly) that puts the wearer at a higher risk of being exposed to the pathogen compared to if it was used perfectly as intended. See detailed description on pages 2 and 3.

158: Was there a minor PPE breach (i.e. skin remains intact)?

In the event where the skin is broken, it constitutes a 'major breach'. See 160.

- Yes: If assessor observes any one of the 7 minor breaches described under item 159 OR if participant reports such an exposure then go to 159.
- No: If assessor does NOT see any of the 7 minor breach types AND the participant does NOT report such exposure.

159: Type of minor breach

If you checked Yes in 158, fill in this section to provide details on the minor breach (i.e. type of breach, tally how many times it happened, place it happened and the time of occurrence).

a) Malposition of face shield

- Yes: If face shield is not centered across and does NOT cover entire face.
- No: If face shield is centered across and covers entire face.

b) Tear in coverall suit (give details where it happened)

- Yes: If assessor observes OR participant reports that there is any breakage in the coverall suit.
- No: If breakage not observed by assessor NOR reported by participant.

N.B: Record the place where the tear was.

c) Tear in gloves (give details where it happened)

This applies to either the outer or the inner pair of glove(s).
- Yes: If assessor observes OR participant reports that there is any breakage in gloves
- No: If breakage not observed by assessor NOR reported by participant

d) Tear in apron (give details where it happened)

- Yes: If assessor observes OR participant reports that there is any breakage in the apron.
- No: If breakage not observed by assessor NOR reported by participant.

e) Attempts to adjust face shield

- Yes: If assessor observes that participant is physically changing the position of the face shield OR participant reports that she/he has physically changed the position of the face shield. (*we need to substantiate the two*)
- No: If assessor does NOT observe that participant is physically changing the position of the face shield AND participant does NOT report that she/he has physically changed the position of the face shield

f) Touches/Scratches any area around the head/face.

- Yes: If assessor observes that participant is physically touching any area around the head/face (i.e. head, face, neck, ears, etc.) OR participant reports doing so.
- No: If assessor does NOT observe that participant is physically touching any area around the head/face (i.e. head, face, neck, ears, etc.) AND participant does NOT report doing so

g) Splash of blood on the PPE

- Yes: If assessor observes that participant has splashes of blood on his/her PPE OR participant reports that she/he got a spill or a splash of blood onto their PPE.
- No: If assessor does NOT observe any splashes on the participant AND participant does NOT report that s/he any spill or splash onto their PPE.

NB: The splashes or the spills should be above the nipple line.

h) Other

- Yes: If assessor observes OR participant reports any other incidents that do not constitute the description of the major breaches mentioned in 161 below. If Yes is checked, record a description of the incident.
- No: If assessor does NOT observe AND participant does NOT report any other incidences.

Record the total number of minor breaches.

160: Was there a major PPE breach (i.e. potential mucous membrane or transcutaneous exposure)?

- Yes: If assessor sees any mucous membrane (i.e. nose, mouth, eyes) exposure to fluids or participant sustains a broken skin (i.e. cut or needle stick injury) OR if participant reports such an exposure. If yes to question 160, then go to 161.
- No: If assessor does NOT see any mucous membrane exposure to fluids or broken skin exposed to the environment AND the participant does NOT report such exposure. If no to question 160, then go to 162

161: Type of major breach.
If you checked Yes in 160 above, fill in this section to provide details on the major breach (i.e. type of breach, tally how many times it happened and the time of occurrence).

a) Needle stick or other sharps injury.
- Yes: If assessor observes OR participant reports any injury caused by needles or other sharp instruments.
- No: If assessor does NOT observe AND participant does NOT report any injury caused by needles or other sharp instruments.

b) Bodily fluid (blood) splash/exposure to skin, eyes and mouth.
- Yes: If assessor observes OR participant reports any direct exposure of his/her mucous membranes and skin with the patient's fluids (i.e. vomit, mucous, urine and blood etc.).
- No: If assessor does NOT observe AND participant does NOT report any contact of the patient's fluids (i.e. vomit, mucous, urine etc.) with their mucous membranes.

c) Contact of the dirty gloves onto the inside of the PPE, skin or mucous membranes
- Yes: If assessor observes OR participant reports any blood splash above the nipple line.
- No: If assessor does NOT observe AND participant does NOT report any blood splash.

d) Other
- Yes: If assessor observes OR participant reports any other incidents that result in exposure of mucous membranes or broken skin. If Yes is checked, record a description of the incidence
- No: If assessor does NOT observe AND participant does NOT report any other incidents that result in exposure of mucous membranes or broken skin

Record the total number of major breaches.

<u>NEAR-MISS INCIDENTS</u>.
162: Was there a near-miss incident (i.e. unsafe sharps practice or tripping over)?
- Yes: If assessor observes OR participant reports any near-miss incident as described in item 163 below. If yes, go to 163.
- No: If assessor does NOT observe AND participant does NOT report any near-miss incident. If no, then go to 164.

163 Type of near-miss incident.
If you checked Yes in 162 above, fill in this section and provide details on the type of near-miss incident (i.e. type of near-miss incident, tally how many times it happened and the time of occurrence).

a) Exposed needle not placed in sharps bin after completion of task.
- Yes: If assessor observes that there is an uncapped needle not placed in a sharps container during or after completion of the task.
- No: If assessor does NOT observe any an uncapped needle not placed in a sharps container during or after completion of the task.

b) Recapping of needle

- Yes: If assessor observes OR participant reports any attempt to replace the cap on an uncapped needle
- No: If assessor does NOT observe AND participant does NOT report any attempt to replace the cap on the uncapped needle.

c) Walking with an exposed needle in the room.

- Yes: If assessor observes OR participant reports moving to different locations in the treatment room while holding an uncapped needle.
- No: If assessor does NOT observe AND participant does NOT move in the treatment room while holding an uncapped needle.

d) Other.

- Yes: If assessor observes OR participant reports any other incidences that may potentially result in a near-miss incident. If Yes is checked, record a description of the incident i.e. if participant drops his/her equipment more than once.
- No: If assessor does NOT observe AND participant does NOT report any other incidents that may potentially result in a near-miss incident.

Record the total number of near-miss incidents.

HEALTH-CONCERNING INCIDENTS.

The next sections (164 to 170) cover health-concerning incidents. In context of this study, as well as functioning in hot climates, the incidents are generally related to symptoms of heat exhaustion. These include, but are not limited to slurred speech, incoherence, dizziness, extreme fatigue, and inability to focus.

164: Was there a health-concerning incident?

- Yes: If assessor observes OR participant reports any health concern while in the chamber or even while doffing or at any time during the study (i.e. feeling extremely tired, dizziness, inability to focus, difficulty breathing).
- No: If assessor does NOT observe AND participant does NOT report any health concern while in the chamber.

165: Did the participant leave the chamber due to this health-concerning incident?

If yes, then record the time when the participant left the chamber. If no, then state the reason why the participant chose to remain in the chamber.

166: Did the participant self-recognize a health concerning incident during the simulation (i.e. exhaustion)?

This section captures the information pertaining to health concerning incidents that the participant voluntarily reports to the study team. The incidents may lead to a decision to remove the participant from the chamber. Always err on the side of being safe. If any member of the study team has even a slight feeling that the participant should be removed from the chamber, inform participant of the decision. If the participant says they would like to be removed from the chamber, assist and facilitate the process immediately.

- Yes: If participant reports any health concern while in the chamber (i.e. feeling extremely tired, dizziness, inability to focus, difficulty breathing).
- No: If participant does NOT report any health concern while in the chamber.
-N/A: If incident did not happen for both the assessor and participant to notice.

Pre-calculated physiological cut-off points.
This study considers 5 physiological cut-offs for every participant that are monitored closely by the study personnel to ensure safety for the participants. These are derived from the heart rate, SBP, DBP, thermal comfort scale level and study personnel assessment of participant (i.e. participant's incoherence to instruction, participant's inability to focus and conduct tasks appropriately and signs of exhaustion from participant etc.).

N.B: If any of the physiological cut-off points are reached, inform the participant and reassess and perform a symptom screen, i.e. 1. Do you feel unwell?, 2. Do you want to continue?, 3. Do you feel light headed?, 4. Do you feel nauseous? etc.

167: Did the participant surpass any of the physiological cut-off points?

The cut-off points are also used in the emergency response protocol. Surpassing any of these cut-offs necessitates that the participant needs to be reassessed before a decision to remove them from the chamber is reached. The *emergency response protocol* has full instructions on what to do in cases of emergency.
- Yes: If any of the participant's physiological parameters (HR, BP, thermal scale rating) exceed the pre-established cut-offs. Go to item 168.
- No: If the participant's physiological parameters (HR, BP, thermal scale rating) do NOT exceed the pre-established cut-offs then go to item 169.

168: What physiological cut-off did the participant surpass? (Check all that apply).

If you checked Yes for 167, record the physiological cut-off(s) that was/were surpassed. Record the time of initial occurrence.

a) 85% of Maximum HR, calculated by the formula of maximum Heart Rate-HRmax = (208-0.7*age) x 0.85.

b) Any HR value that deviates from 30-200 beats/minute necessitates a safety screen of the participant. N.B: for every participant use HRmax as their upper limit to prompt a screen

c) A systolic BP of less than 90 mmHg on two successive readings taken 3-5 minutes apart.

d) A drop of greater than 40 mmHg in SBP from the baseline value.

e) A rise of greater than 180 mmHg in SBP.

169: Was participant removed from the simulation for any other reason? OR was there any other incident that caused the participant to be removed?

If participant was not removed from the simulation for any of the reasons mentioned in 164, 165 and 168 or for any other reason, then the form is complete. If yes to 169, then proceed to 170.

170: Other types of incidents: Record all the times any of these happens

a) Assessor's discretion (e.g. participant couldn't focus, or conduct tasks appropriately, or was incoherent with instruction, etc.)
- Yes: Assessor's discretion, note down the reason.
- No: If none of the above happens.

b) The participant did not want to continue participating the study.
- Yes: If the participant chooses to terminate their participation in the study for any other reason not captured above. This could be during the donning, doffing or orientation or at any point during the study. State the reason the participant gave for not continuing with the study
- No: If none of the above happens.

c) If participant has reached a thermal scale of ≥12 or any scale individualized to the participant.
N.B: This is relative to the participant's perception of their heat comfort scale. If the participant exits the chamber as a result of the heat strain, then note the time and their thermal scale.

d) If participant has reached a thermal scale of ≥ 12 and they choose not to exit the chamber after informing them, then note the reason as to why the stayed in the chamber.

e) Any other incident that could have caused the experiment to end.

Numbers that do not attract a score for the participant.

These numbers do not attract scores for participants; 1-10, 23-28, 52-54, 102-104, 152-157 and 158-170.

Additional Notes:
-Record any other information regarding any incident that occurred that is not captured in the CRF.
- Note any other comments from the participants.
